# Dynamic changes and improvement paths of China’s emergency logistics response capabilities under public emergencies—research based on the entropy weight TOPSIS method

**DOI:** 10.3389/fpubh.2024.1397747

**Published:** 2024-07-10

**Authors:** Heng Chen, Yuan Guo, Xianglong Lin, Xianchao Qi

**Affiliations:** School of Management, Xi’an Polytechnic University, Xi’an, China

**Keywords:** public emergency shock, emergency logistics components, emergency logistics response capability, enhancement paths, Chinese samples

## Abstract

**Introduction:**

Due to global industrialization and urbanization, natural disasters, accidents, and public health emergencies happen frequently. These events cause significant loss of life and property damage to countries worldwide. In the context of frequent public emergencies, enhancing emergency logistics response capabilities is crucial, ensuring rapid supply of rescue materials and support for rescue personnel, thereby saving lives and reducing economic losses.

**Methods:**

In order to identify the changes and enhancement paths of the emergency logistics response capability of Chinese regions under the shocks of public emergencies, this paper innovatively constructs emergency logistics response capability measurement indicators. This paper uses the entropy weight TOPSIS method and panel quantile regression model to quantify the change and enhancement paths of China’s regional emergency logistics response capability under different events.

**Results:**

It is found that: (1) The gap in emergency logistics response capability among Chinese regions is widening, with the internal difference in the eastern region higher than that in the west, while the difference in the central region is relatively low. (2) China’s emergency management department can effectively transform social logistics into emergency logistics, thereby promoting the improvement of emergency logistics response capabilities. (3) Sudden geological disasters break through the limits of social logistics resources when they cross lower scales, resulting in the failure of emergency logistics response capabilities.

**Discussion:**

This paper expands research on assessing emergency logistics capabilities, addressing issues in existing assessments such as reliance on single indicators and subjective measurement methods. Additionally, it quantifies the dynamic changes in China’s regional emergency logistics response capabilities under public emergencies by extending the study of event content, types, and impacts. This enhances discussions on the effects of public emergencies. Finally, from an empirical perspective, the paper explores pathways to enhance regional emergency logistics response capabilities in China. In practice, this paper assists countries worldwide in assessing whether different regions of China can effectively provide emergency support for various resources in direct investments, thus providing a scientific basis for investment decisions.

## 1 Introduction

With the accelerated industrialization and urbanization of the world, as well as the deterioration of the atmospheric and geological environments, a variety of natural disasters, accidents, catastrophes and public health events have occurred frequently. This has caused huge casualties and heavy property losses to countries around the world. Compared with most countries, China is one of the few countries with the most serious natural disasters. According to the data for 2020, China was affected by public emergencies, causing 138 million victims and direct economic losses amounting to 370.145 billion yuan, accounting for 0.375% of GDP. Therefore, how to closely prevent and effectively respond to the losses caused by various types of public emergencies. This has become a matter of close attention for countries around the world. Moreover, Domestic and foreign governments and academia generally believe that “building a hierarchical response system for emergency logistics is an important safeguard and a powerful support for preventing and responding to public emergencies.”

Under the onslaught of frequent public emergencies, it is vital to improve emergency logistics response capabilities. This can ensure the rapid supply of relief materials and support for rescuers, thus saving lives and mitigating economic losses. Therefore, research on emergency logistics has received keen attention from academics, governments and the public. However, although scholars have studied the connotation, types and impacts of public emergencies, the existing studies have only analyzed the connotation and types of public emergencies and their impacts on economic growth from a qualitative perspective, and they lack quantitative studies. Secondly, scholars have studied the model of emergency logistics, but mainly from the construction of mathematical and theoretical models and simulation points of view on the emergency logistics model and site selection, there is a lack of research on the emergency logistics response capability from a regional perspective. Finally, although scholars have evaluated the emergency logistics response capability. However, most of the evaluation methods are subjective assignment methods or mathematical analysis methods, which cannot avoid the influence of subjective human factors on the evaluation results. Thus reduced the scientific nature of the measurement results and led to the limited reliability of the evaluation results. Based on this, the research objectives of this paper are: (1) Measure the emergency logistics response capability of Chinese regions and analyze the regional differences. (2) Explore the dynamic changes in China’s emergency logistics response capability under the impact of public emergencies through empirical research. (3) Combined with the selection of factors to enhance emergency logistics response capability, the key path to enhancing China’s emergency logistics response capability is analyzed using empirical methods.

## 2 Literature review

Considering the study’s objectives and themes, along with the relevant literature from both domestic and international sources, it is evident that existing research has primarily focused on three main areas. Specifically, as follows:

### 2.1 Research on the connotation, types and impacts of public emergencies

As an important factor that jeopardizes economic growth and social security, public emergencies have triggered scholars to study the connotation, type and impact of public emergencies.

Research on connotation of public emergencies: Xue and Liu (1) define public emergencies as sudden events that cause or may cause serious social harm, threaten public order, and result in significant casualties, property losses, and damage to the ecological environment. Western scholars often use the term “crisis” to describe similar public emergencies. According to Uriel et al. (2), a crisis is an event that poses a serious threat to social systems, requiring critical decisions to be made within a short time frame and under high uncertainty. Exum et al. (3) believe that “public emergencies” in the United States are mostly characterized as “emergency affairs,” which in the field of public management can be summarized as events that may pose a threat to people’s lives, public health security, or property security.

Research on types of public emergencies: According to Mlađan and Cvetković (4), all emergencies can be classified into three framework types: natural emergencies, emergencies that are related (directly or indirectly) to human beings (they are called anthropogenic, technological, technical-technological, social) and hybrid emergencies (combination of acts of natural forces and the influence of human decisions). Xue and Liu (1) classified public emergencies into natural disasters, public health events, accident disasters, and social security events based on their occurrence process and nature.

Research on impact of public emergencies: The research on the impact of public emergencies mainly focuses on the impact of natural disasters and public health events on social and economic growth. Taking natural disasters as an example, Li (5) found that natural disasters caused a large amount of capital to be destroyed, and by affecting production factors, it may hurt economic growth in both the long and short term. Regarding public health events, Kostova et al. (6) pointed out through research that the impact of various public health emergencies will not have a long-term impact on the macroeconomy, and the macroeconomy negatively affected by the epidemic can usually recover quickly within one year. Ceylan and Ozkan (7) assessed the impact of SARS-COV and MERS-COV on macroeconomic conditions, income levels, and labor market composition in 26 selected countries. Antràs et al. (8) mentioned in their research that the rapid spread of COVID-19 around the world has brought tremendous financial pressure to governments in various countries and regions and, at the same time, increased uncertainty in various markets and risks in financial markets.

### 2.2 Model research on emergency logistics

The emergency logistics model is a planning program that utilizes demand forecasting to provide a complete platform and application method for emergency logistics distribution. Its purpose is to ensure the accurate allocation of emergency supplies and the exact matching of supply and demand. As a result, many scholars have conducted research on the emergency logistics model. For example, Rasouli (9) and Rodríguez-Espíndola et al. (10) utilized emerging technologies, such as intelligent sensing systems and integrated disruptive technologies, to develop an emergency logistics model for enhancing the efficiency of emergency material distribution. Rodríguez-Espíndola et al. (10) integrated three emerging disruptive technologies, namely artificial intelligence, blockchain, and 3D printing, in order to improve the efficiency of the emergency material distribution model. Lu and Luo (11) proposed a novel emergency logistics model that simulates the emergency transportation scenarios from the logistics center to each disaster point and between each disaster point. Ghelichi et al. (12) proposed a simulation-based performance evaluation model for the drone-based delivery of aid items to disaster-affected areas.

However, the distribution of emergency supplies may expose various issues, including imperfect dispatching mechanisms, delayed response, and disconnect between supply and demand. Therefore, the key to effective disaster relief lies in designing the distribution network, implementing dynamic dispatching, and optimizing the reasonable distribution of emergency supplies. Jeong et al. (13) provided an integrated framework to design emergency logistics networks (ELNs) based upon efficiency, risk and robustness metrics. Alem et al. (14) proposed a new dynamic two-stage stochastic network flow model for disaster relief.

After designing the emergency logistics network, the next step is to dispatch emergency supplies dynamically. For example, Manopiniwes and Irohara (15) developed an integrated decision-making model that considers three key areas of emergency logistics: facility and stock prepositioning, evacuation planning and relief vehicle planning. Boonmee et al. (16) used a combination of precise and heuristic algorithms to select the location of emergency logistics facilities. Maharjan and Hanaoka (17) developed a multi-objective location-allocation model for relief supply and distribution. In addition, Ransikarbum and Mason (18) believed that through geographic information systems (GIS), emergency logistics managers can quickly obtain and analyze critical geospatial data to make timely and informed decisions in disaster response.

In addition, the following research difficulty is how to carry out reasonable allocation. To this end, different scholars have completed research on emergency logistics resource allocation and decision-making by building optimization models. Loree and Aros-Vera (19) developed a mathematical model to determine the location of distribution points (PODs) and inventory distribution in post-disaster humanitarian logistics (PD-HL). The model minimizes facility placement, logistics, and deprivation costs. The super network model proposed by Yuan and Wang (20) addressed the research gap of only considering the emergency logistics supply chain provides a solution for emergency material dispatching and achieves the reasonable distribution and optimization of disaster relief materials.

### 2.3 Emergency logistics capability evaluation study

Emergency logistics response capability, as an important part of emergency management, not only runs through all stages of emergency response, but also is a powerful support for coping with public emergencies. Thus, many scholars have done a lot of research on the evaluation of emergency logistics response capability. For example, Sheu (21) used a combination of fuzzy clustering, entropy method and TOPSIS method to analyze the dynamic rescue needs of emergency logistics under disaster conditions. Moltchanova et al. (22) developed a stochastic model to evaluate the economic losses and loss of life to assist efficient earthquake response. Nachtmann and Pohl (23) provided a scorecard based on the principles of value-centered thinking for evaluating transportation preparedness in emergency action plans. Ma et al. (24) constructed a maturity model of emergency logistics response capability, which mathematically shows that this system can effectively judge the maturity of emergency logistics response capability. Jiang and Yuan (25) constructed an evaluation system of emergency logistics guarantee capability under sudden infectious epidemics based on the hybrid MADM model. Zhang et al. (26) analyzed the public health emergency logistics capability of the COVID-19 epidemic based on the emergency logistics capability evaluation model of BP neural network.

### 2.4 Research gaps

By combing and summarizing the existing research results. In terms of the research on the connotation, types and impacts of public emergencies, the existing research has classified public emergencies, which provides inspiration for the research in this paper. However, existing research lacks quantitative research on the impact of public emergencies. Emergency logistics has the potential to reduce harm in public emergencies, but its effect depends on the strength of emergency logistics response capability. However, existing studies have paid little attention to this issue. In terms of emergency logistics model optimization and site selection, the main focus has been on the construction and simulation of mathematical and theoretical models, but there is no research involving different regional emergency logistics response capabilities and their resilience. In order to improve the emergency logistics response capability, it is necessary to construct a hierarchical response system, which needs to be based on research on emergency logistics response capability and its toughness. In the evaluation of emergency logistics response capability, some scholars have conducted evaluations, but the methods mostly favor the subjective assignment method or mathematical analysis method, which reduces the scientificity of the measurement results. In addition, existing studies pay less attention to the path of how to improve emergency logistics response capability. Therefore, the research in this paper was extended as follows: (1) A comprehensive index system was constructed to measure the emergency logistics response capability in different regions of China. This system combines various factors related to emergency logistics and uses the entropy weight TOPSIS method for measurement and analysis. (2) By constructing the regression method of the number of panel division, under the impact of emergencies and its classification events, this article analyzes the dynamic changes of China’s emergency logistics response capability. (3) On the basis of analyzing the key factors for the improvement of emergency logistics response capability, through building a panel regression model, the path of enhancement of emergency logistics response capability was inspected. The research gaps in this article are shown in Table 1.

**Table 1:** Research gaps.

Research direction	Existing research	This research
Research on the connotation, type and impact of public emergencies	Only classify public emergencies, there is a lack of quantitative research on the impact of public emergencies; Less attention is paid to the strength of emergency logistics response capabilities under the influence of public emergencies.	Further conduct quantitative research on the impact of public emergencies; analyze the dynamic changes in China’s emergency logistics response capabilities under the impact of public emergencies and their classified events.
Emergency logistics model optimization and site selection research	It mainly focuses on constructing or simulating mathematical models and lacks research on the emergency logistics response capabilities and resilience of different regions.	Combining emergency logistics response capabilities and resilience, building a hierarchical response system, optimizing models and site selection plans.
Emergency logistics response capability evaluation	Most methods focus on subjective assignment methods or mathematical analysis, lacking scientific measurement; Less attention is paid to improving the path of emergency logistics response capabilities.	A comprehensive measurement index system and a panel regression model were constructed to empirically test the improvement path of emergency logistics response capabilities.

## 3 Measurement of regional emergency logistics response capability and analysis of regional differences in China

### 3.1 Components of emergency logistics response capability

Emergency logistics is a special logistics activity used to respond to emergencies such as natural disasters, public health crises, accidental catastrophes, security incidents and military conflicts. Emergency logistics, like social logistics, is composed of fluid carriers, flow direction, flow rate and other elements (27). According to Kołodziejczyk and Szołtysek (28), social logistics can be defined as the art of managing socially significant material flows (and related information flows) in order to achieve certain spatial and temporal advantages necessary to ensure the proper functioning of society and to provide adequate quality of life. The relationship between emergency logistics and social logistics is reflected in the occurrence of public emergencies, and social logistics needs to be transformed into emergency logistics to meet the demand for emergency supplies. That is, emergency logistics is the whole logistics activity in terms of response time, response speed, emergency material transportation quality, emergency material delivery punctuality, delivery reliability and other aspects of the comprehensive emergency response capability. The distinction between social logistics and emergency logistics is illustrated in Table 2.

**Table 2:** The difference between social logistics and emergency logistics.

Elements	Social logistics	Emergency logistics
Final goal	Profit	Improve rescue efficiency
Carrying supplies	Trading commodities and goods	Disaster relief supplies
Means of transport	Third-party logistics fleet, etc.	Emergency fleets for third-party logistics, military and other units
Demand	Long term stability	Rapid increase in short term
Flow direction	Market demand customers	Needs of people in disaster areas
Flow speed	Stable shipping time	Impact of road conditions in disaster-stricken areas

In the study of logistics capability, scholars have reached a consensus on the metrics of logistics carrier, flow rate and flow efficiency. Based on these elements, this paper builds an index system including emergency logistics carrier capability, emergency logistics flow capability, emergency logistics flow rate capability, and emergency logistics flow efficiency capability based on the analysis of emergency logistics capability components and index selection by Xi (29). This study considers that social and medical resources are the government’s responsibility during public emergencies and form part of emergency logistics capabilities. Drawing on the research of Bao-de et al. (30) research, an index system related to social emergency guarantee capabilities is introduced. In addition, Glick (31) believes that informationization capability is an essential factor affecting the speed of change in logistics capabilities. Therefore, indicators related to regional informatization capabilities are further introduced as components of emergency logistics response capabilities.

In the selection of sample data, considering the availability of data, the sample observed in this paper contains 30 provinces and regions in China (excluding Tibet, Hong Kong, Macao and Taiwan); the sample observation period is 2012–2021, and the data are all obtained from the China Statistical Yearbook and the website of the National Bureau of Statistics.^1^ Among them, missing values are replaced by interpolation or analogy, and some indicators are calculated from the original data. The composition of the specific indicator system is shown in Table 3.

**Table 3:** Components of emergency logistics response capability factors and indicator system.

First level indicators	Second level indicators	Third level indicators	Indicator properties
Emergency logistics carrier carrying capability	Logistics infrastructure	Transportation network density	+
	Ownership of logistics and transportation equipment	Civilian vehicle ownership	+
		Vehicle ownership for road operations	+
	Logistics industry practitioners	Employment in rail, road, air, stevedoring and other transportation, warehousing and postal services	+
	Logistics carrier capability	Express business volume	+
Emergency logistics flow capability	Volume of freight	Railroad freight	+
		Road freight	+
	Freight turnover	Railroad freight turnover	+
		Road freight turnover	+
Emergency logistics flow rate capability	Share of graded roads	Percentage of highways	+
		Percentage of primary roads	+
		Percentage of secondary roads	+
Emergency logistics Flow effectiveness capability	Volume of material flows completed per unit of manpower	Freight volume to employment ratio	+
		Freight turnover to employment ratio	+
	Flow of goods per unit of capital investment completed	Ratio of freight volume to capital investment in the logistics industry	+
		Ratio of freight turnover to capital investment in the logistics industry	+
Regional informatization capability	Volume of telecommunication services	Total postal and telecommunication operations	+
	Network infrastructure	Fiber optic line length	+
	Level of regional communications	Number of cell phone subscribers	+
		Number of Internet broadband access ports	+
Social emergency guarantee capability	Rescue guarantee foundation	Number of centers for disease control and prevention	+
		Number of health technicians	+
		Number of beds in health facilities	+
	Regional policy support	Share of local transport expenditures in total expenditures	+

First-level indicators reflect six core aspects of emergency logistics response capability: carrier carrying capability, flow capability, flow rate capability, flow efficiency capability, regional information capability and social emergency guarantee capability. First-level indicators are the main basis for assessing emergency logistics performance. The second-level describe in detail the components of the primary indicators. For example, the carrying capability of emergency logistics carriers can be further subdivided into indicators such as logistics infrastructure, ownership of logistics transportation equipment, employees in the logistics industry and logistics carrier capability. Similarly, other first-level indicators have corresponding second-level. Third-level indicators quantify the second-level at a more specific level; For example, the ownership of logistics transportation equipment can be further divided into the ownership of civilian vehicles, highway operating vehicles and other specific attributes. Similarly, other second-level contain corresponding tertiary indicators. All of these indicators have positive attributes, indicating that they are positive, beneficial and excellent in assessing the emergency logistics response capability. For example, a higher density of the transportation network, a higher ownership of logistics and transportation equipment, or a higher volume of freight transport all indicate a higher emergency logistics response capability.

### 3.2 Model for measuring emergency logistics response capability

Entropy weight TOPSIS method is a combination of entropy weight method and TOPSIS method, which is a kind of target decision analysis method used for comprehensive level assessment of multiple programs. The advantage of entropy weight method is to standardize the indicators and objectively determine the weights according to the degree of variability to avoid human interference; the advantage of TOPSIS method is to quantify the ranking by comparing the size of the relative distance between the evaluation object and the ideal point. In view of this, this paper adopts the entropy weight TOPSIS method to measure the emergency logistics response capability in 2012–2021, and its core idea is based on the standardization of each measurement index. The entropy weight method is used to give the weight value of each measurement index, and then quantitatively rank the emergency logistics response capability of each province using the TOPSIS method. The specific calculation steps of the method are as follows:

1. Standardized processing of raw data. In order to facilitate the comparison of the indicators of different provinces in each year. Therefore, this article performs dimensionless processing of the data and finally obtains the measurement value of the emergency logistics response capability of each province in China. The indicators selected in this paper are all positive indicators. The data processing is shown in equation 3-1:

(3-1) $$x_{\theta ij}^{\prime }=\frac{x_{\theta ij}-\min\left\{x_{\theta 1j},\ldots ,x_{\theta nj}\right\}}{\max\left\{x_{\theta 1j},\ldots ,x_{\theta nj}\right\}-\min\left\{x_{\theta 1j},\ldots ,x_{\theta nj}\right\}}$$

Where: assuming *r* years, *n* provinces (districts, cities), *m* indicators, then $x_{\theta ij}$ represents the value of the *j-th* indicator of province (district, city) *i* in year *θ*, of which θ=1,2,…,r;i=1,2,…,n;j=1,2…,m _._ $x_{\theta ij}$ is the original value (initialized value) of the indicator; $x_{\max}$ and $x_{\min}$ are the maximum and minimum values of the group where the indicator is located; $x_{\theta ij}^{\prime }$ is the standardized value of the indicator.

1. Calculation of weights
2.
$p_{\theta ij}$ indicates the weight of the *i-th* program under the *j-th* indicator for that indicator, as shown in equation 3-2:

(3-2) $$p_{\theta ij}=\frac{x_{\theta ij}^{\prime }}{\sum_{\theta =1}^{r}\sum_{i=1}^{n}x_{\theta ij}^{\prime }}$$

1. Find the information entropy of the *j-th* indicator $e_{j}$ , as shown in equation 3-3:

(3-3) $$e_{j}=-\frac{\sum_{\theta =1}^{r}\sum_{i=1}^{n}\left(p_{\theta ij}*\ln p_{\theta ij}\right)}{\ln rn}$$

1.
$w_{j}$ denotes the weight of the *j-th* indicator, as shown in equation 3-4:

(3-4) $$w_{j}=\frac{1-e_{j}}{\sum_{j=1}^{n}\left(1-e_{j}\right)}$$

1. Use the weights determined based on the entropy weight TOPSIS method $w_{j}$ to create the matrix *Z*

$$\begin{array}{l} Z={\left(z_{\theta ij}\right)}_{r\times m\times n},z_{\theta ij}=w_{j}\times x_{\theta ij}^{\prime }\\ \left(\theta =1,2...,r,i=1,2...,n;j=1,2,...,m\right) \end{array}$$

$z_{\theta ij}$ denotes the weighted decision score; *Z* is the weighted decision matrix consisting of all the weighted decision scores.

1. Calculate the Euclidean distance $D_{\theta i}+$ and $D_{\theta i}-$ between each solution and the positive ideal solution $d_{j}+$ and the negative ideal solution $d_{j}-$ , as shown in equations 3-5, 3-6

(3-5) $$D_{\theta i}+=\sqrt{\overset{n}{\underset{j=1}{\sum }}{\left(d_{j}+-z_{\theta ij}\right)}^{2}}\left(\mathrm{math}.\right)\;\mathrm{genus}\;d_{j}+=\max\left(z_{\theta ij}\right)$$

(3-6) $$D_{\theta i}-=\sqrt{\overset{n}{\underset{j=1}{\sum }}{\left(d_{j}--z_{\theta ij}\right)}^{2}}\left(\mathrm{math}.\right)\;\mathrm{genus}\;d_{j}-=\min\left(z_{\theta ij}\right)$$

1. Calculation of emergency logistics response capability $C_{\theta i}$ , as shown in equation 3-7:

(3-7) $$C_{\theta i}=\frac{D_{\theta i}-}{D_{\theta i}++D_{\theta i}-}$$

The closer the $C_{\theta i}$ value is to 1, the closer the program is to the relative distance from the ideal sample solution, and the higher the emergency logistics response capability of the region, and the closer the $C_{\theta i}$ value is to 0, the lower the emergency logistics response capability of the region.

### 3.3 Convergence analysis of emergency logistics response capability

This paper adopts α convergence to study the convergence of regional emergency logistics response capability. α convergence is generally measured by the coefficient of variation, and if there is a tendency of shrinking of the national and three regions’ emergency logistics capability, it means that there is α convergence. The specific equation is shown in 3-8:


(3-8) $$K=\sqrt{\frac{\overset{n}{\underset{i=1}{\sum }}{\left(X_{i}-\mu \right)}^{2}}{n}}/\mu$$

Where *i* denotes the *i-th* province (i = 1,2,3,…,*N*), *n* is the number of province, $x_{i}$ is the emergency logistics response capability of the *i-th* province, μ denotes the mean value of the emergency logistics response capability, and *K* represents the coefficient of variation. If the value of *K* gradually decreases during the sample period, it indicates that there is α convergence of emergency logistics response capability.

### 3.4 Analysis of calculation results

#### 3.4.1 Measurement results of China’s regional emergency logistics response capability

Based on the above index system, this paper applies Stata software to measure the emergency logistics response capability of 30 Chinese provinces (autonomous regions and municipalities directly under the central government) from 2012 to 2021 according to the entropy weight TOPSIS method. The weight results of the measurement indicators calculated by the entropy value method are shown in Table 4. The emergency logistics response capability measurement results are shown in Table 5. In order to more comprehensively grasp the development dynamics of regional emergency logistics, this paper refers to the division of China’s regions by China’s National Development and Reform Commission. China’s 31 provinces are divided into three major economic regions of the east, the center and the west,^2^ and the emergency logistics response capability of the three major economic regions is measured and analyzed.

**Table 4:** Summary of weight calculation results using entropy value method.

Indicators	Information entropy value *e*	Information utility value *d*	Weight coefficient *w* (%)
Transport network density	0.9528	0.0472	3.60
Civilian vehicle ownership	0.9517	0.0483	3.69
Number of highway operating vehicles	0.9634	0.0366	2.79
Number of employees in railways, highways, aviation, loading and unloading and other transportation, warehousing, and postal services	0.9695	0.0305	2.33
Express delivery volume	0.7918	0.2082	15.90
Railway freight volume	0.8915	0.1085	8.29
Road freight volume	0.9603	0.0397	3.03
Railway freight turnover	0.8931	0.1069	8.16
Highway freight turnover	0.9343	0.0657	5.02
Highway share	0.9899	0.0101	0.77
Proportion of first-class highways	0.9441	0.0559	4.27
Secondary roads	0.9833	0.0167	1.28
Freight volume to employment ratio	0.975	0.025	1.91
Ratio of freight turnover to employed persons	0.9661	0.0339	2.59
Ratio of freight volume to capital investment in logistics industry	0.9583	0.0417	3.18
Ratio of freight turnover to capital investment in logistics industry	0.911	0.089	6.80
Postal and telecommunications business volume	0.8926	0.1074	8.20
Length of optical cable line	0.9525	0.0475	3.62
Number of mobile phone users	0.9605	0.0395	3.01
Number of Internet broadband access ports	0.9505	0.0495	3.78
Number of Centers for Disease Control and Prevention	0.9751	0.0249	1.90
Number of health technicians	0.9672	0.0328	2.51
Number of beds in health institutions	0.9659	0.0341	2.60
The proportion of local fiscal expenditure on transportation in total expenditure	0.9897	0.0103	0.78

**Table 5:** Results of China’s emergency logistics response capability measurement by region.

Vintages	Eastern part	Central part	Western part
2012	0.19583	0.171711	0.111709
2013	0.21041	0.174922	0.118536
2014	0.22095	0.179956	0.122255
2015	0.22523	0.176467	0.122782
2016	0.23526	0.183678	0.126145
2017	0.25276	0.199089	0.134827
2018	0.27286	0.211689	0.146955
2019	0.29071	0.210078	0.147945
2020	0.3133	0.215867	0.154155
2021	0.33364	0.222533	0.160982

**Figure 1:**
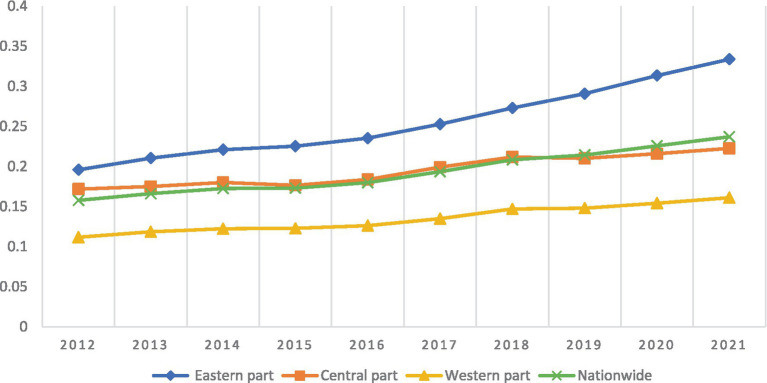
Evolution of China’s emergency logistics response capabilities from 2012 to 2021.

This study analyzes the trend of emergency logistics response capability by region in China (see Figure 1). At the national level, China’s emergency logistics response capability improved significantly during the sample observation period. The national average increased from 0.1577 in 2012 to 0.2370 in 2021, an increase of 50.29%. Among them, the growth rate in 2017–2018 was the highest. The possible reason is that the country established the Ministry of Emergency Management in 2018. The Ministry of Emergency Management continues to issue corresponding plans and regards emergency logistics as a key project for construction and comprehensive deployment. Thus rapidly improving China’s emergency logistics response capability. At the regional level, the emergency logistics response capability in eastern, central, and western China has been steadily increasing, in the order of eastern > central > western regions. The gap in emergency logistics response capability between different regions is mainly due to the following reasons: Firstly, China has diverse geographic and climatic conditions, and the scale, type and severity of public emergencies faced by different regions vary greatly. Secondly, the sharing system among relevant departments is not yet complete, and an emergency information data sharing platform for different regions has not yet been established. It is temporarily unable to give full play to the advantages of relevant departments such as the Ministry of Emergency Management and the National Grain and Material Reserves Bureau. This has resulted in an imperfect cross-regional allocation mechanism for emergency supplies, making it impossible to jointly promote the synergy of emergency logistics response capability in different regions. Finally, there are differences in the government’s early warning and protection capabilities in different regions, including the size of the Centers for Disease Control and Prevention (CDCs), the size and quality of medical resources, and local financial investment in emergency logistics. This results in differences in the emergency logistics response capabilities of different regions in China.

#### 3.4.2 Convergence analysis of China’s regional emergency logistics response capability

Through the convergence analysis of China’s regional emergency logistics response capability, the results are shown in Tables 6, 7. From the results in Table 5, It can be obtained that, in terms of the difference in emergency logistics response capabilities of the provinces under its jurisdiction, the eastern region > the western region > the central region. Further by calculating the variability of emergency logistics response capability between the eastern, central and western regions, as shown in Table 6, it can be seen that the variability of emergency logistics response capability between the eastern and central-western regions is increasing. Based on the average measurement of emergency logistics response capabilities in the eastern, central, and western regions. The eastern-central, eastern-western, and central-western samples were assembled, and the α convergence model was used to calculate the differences. This obtained the variation trend of differences between the eastern-central, eastern-western, and central-western samples. As shown in Table 7, the variability in the East–West is higher than that in the East-Central region.

**Table 6:** China’s emergency logistics response capability nationally and within regions α convergence results.

Vintages	Nationwide	Eastern part	Central part	Western part
2012	0.41521	0.337765	0.329009	0.34835
2013	0.443692	0.409443	0.296235	0.353259
2014	0.449756	0.404369	0.305689	0.363218
2015	0.459255	0.414408	0.307494	0.353385
2016	0.474335	0.427979	0.319796	0.363759
2017	0.482235	0.442157	0.307746	0.381082
2018	0.494704	0.462878	0.316496	0.387088
2019	0.505924	0.450264	0.318834	0.373211
2020	0.519843	0.450308	0.320705	0.375986
2021	0.539681	0.467788	0.321588	0.38504

**Table 7:** Convergence results between regions of China’s emergency logistics response capability α .

Vintages	Eastern-central	Central-western	Eastern-western
2012	0.341231	0.405272	0.44891
2013	0.382795	0.378028	0.499011
2014	0.386074	0.385822	0.503554
2015	0.401086	0.377306	0.513204
2016	0.414413	0.390786	0.529692
2017	0.418924	0.394048	0.544132
2018	0.438846	0.394964	0.556222
2019	0.447405	0.387702	0.564161
2020	0.460173	0.386613	0.576225
2021	0.481711	0.387858	0.596539

## 4 Dynamic changes in China’s regional emergency logistics response capability under the shock of public emergencies

Combined with the above analysis of China’s regional emergency logistics response capability measurement and its convergence, it is found that the difference in China’s regional emergency logistics response capabilities is in an expanding trend. Then, what is the potential response capability of China’s emergency logistics under the impact of public emergencies? To address this question, an empirical study is conducted in this part.

### 4.1 Indicator selection and data sources

#### 4.1.1 Dependent variable selection

Based on the research objectives, this paper selects emergency logistics response capability (*logistic*) as the dependent variable. The sample observation period is 2012–2021. The underlying data comes from the results measured by the entropy weight TOPSIS method in Section 3.1 of this paper.

#### 4.1.2 Selection of core variables

The purpose of this study is to analyze the dynamic changes in emergency logistics response capability under the impact of public emergencies. We selected the scale of public emergencies (accident) as the core explanatory variable. The observation period of the sample is from 2012 to 2021, and the sum of the number of geological disasters, fires, and environmental emergencies is used as a proxy variable. In addition, in order to deeply study the impact of different types of public emergencies on emergency logistics response capability. We categorize public emergencies into three types: the number of geologic disasters, the number of fires, and the number of environmental emergencies. The following examines the dynamic changes in emergency logistics response capabilities caused by these three types of events. The data for the study comes from the yearbook of the National Bureau of Statistics of China, and the sample observation period is 2012–2021. For definitions and descriptions of specific variables, please refer to Table 8.

**Table 8:** Definitions and descriptions of variables.

Variable type	Variable name	Variable symbol	Indicator measurement
Implicit variable	Emergency logistics response capability	*logistic*	Results measured using the entropy weight TOPSIS method.
Core variable	Scale of public emergencies	*accident*	The sum of the number of occurrences of geologic hazards, the number of fires, and the number of environmental emergencies were used as proxy variables.
	Scale of sudden-onset geologic disasters	*geology*	Number of geologic hazards.
	Scale of fire outbreaks	*fire*	Number of fires.
	Scale of environmental emergencies	*environment*	Number of environmental emergencies.

### 4.2 Model construction

Based on the research objectives and indicator selection, this paper chooses *logistic* as the dependent variable and *accident, geology, fire, and environment* as the independent variables. By constructing a panel quantile panel regression model, this paper explores the dynamic changes in emergency logistics response capabilities under the impact of large-scale public emergencies. As shown in model 4-1, the specific form is shown below:


(4-1) $$lnlogistic=\alpha_{1q}+b_{2q}lnaccident_{tq}+\delta_{t}$$

(4-2) $$\begin{array}{l} lnlogistic=\alpha_{1q}+b_{2q}lngeology_{tq}+b_{3q}lnfire_{tq}\\ \;\;\;\;\;\;\;\;\;\;\;\;\;\;\;\;\;\;\;\;\;\;\;\;+b_{4q}lnenvironment_{tq}+\delta_{t} \end{array}$$

In models 4-1 and 4-2, lnlogistic represents the emergency logistics response capability; $lnaccident_{tq}$ represents the scale of public emergencies at different quartiles (0.25, 0.5, 0.75 quartiles are selected in this study); $lngeology_{tq}$ , $lnfire_{tq}$ , $lnenvironment_{tq}$ represent the scale of geologic disasters, fire emergencies, and environmental emergencies (0.25, 0.5, 0.75 quartiles are selected in this study); $\delta_{it}$ represents the random perturbation term; *it* represents the sample of a province (autonomous region, municipality directly under the central government) in China at a certain time from 2012 to 2021.

### 4.3 Test of empirical results

#### 4.3.1 Impact of shocks to the overall scale of public emergencies

**Figure 2:**
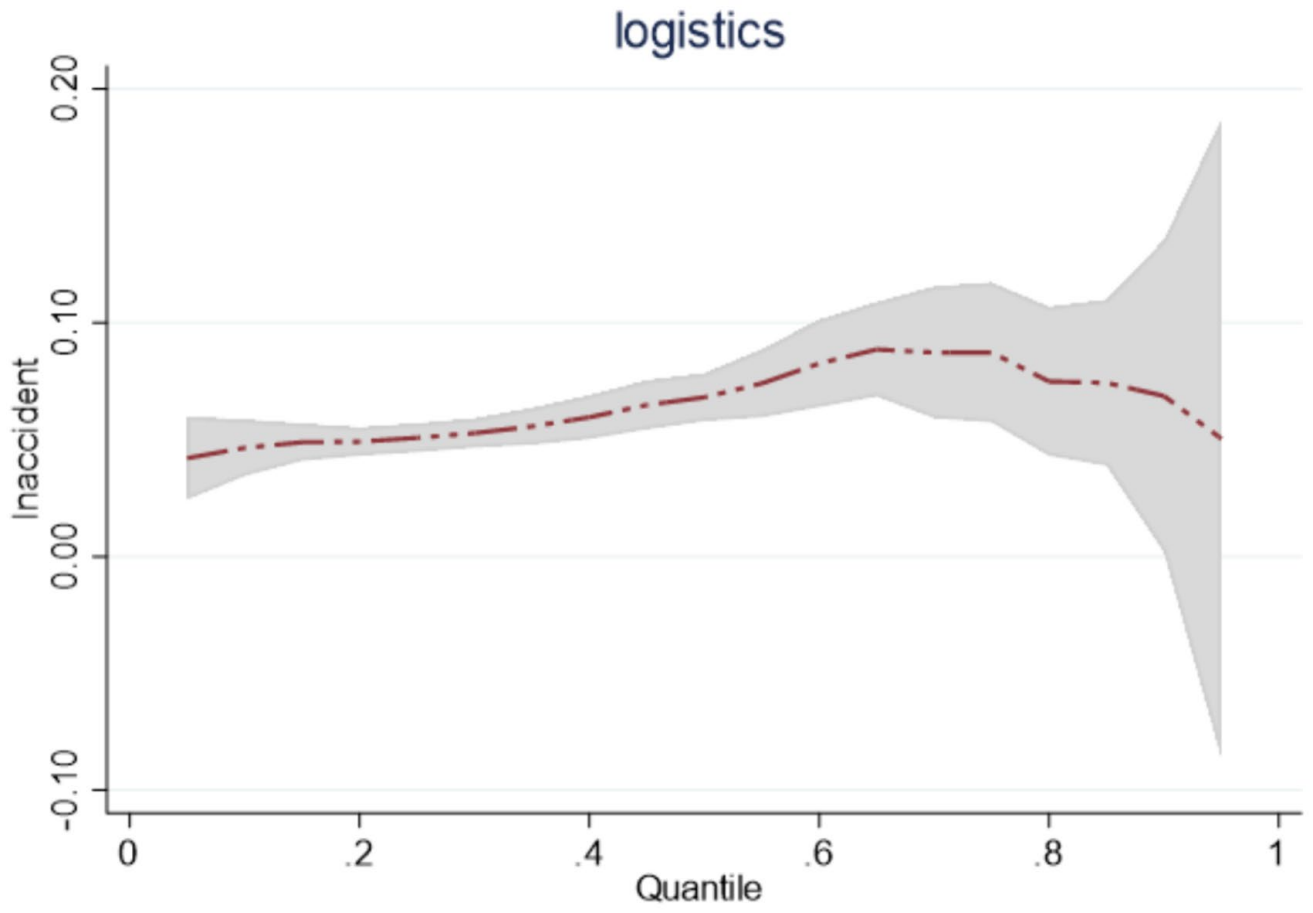
Dynamics of China’s emergency logistics response capability under the impact of a national sample of public emergencies.

Considering that the panel data analyzed in this paper are short panels, the smoothness and cointegration tests can be excluded from the regression. Based on the panel quantile regression model with a semi-logarithmic model (4-1), this paper conducts the quantile regression for the national sample, the eastern region, the central region, and the western region sample, and the regression results are shown in Table 9. Observed from the national sample, the positive regression coefficients of *lnaccident* at quantiles 0.25, 0.5, 0.75 are all significant, and the change of the impact effect on *lnlogistics* continues to improve from 0.0507 to 0.08721, indicating that with the expansion of the scale of the public emergency, China’s emergency logistics response capability continues to improve. However, from the impact trend, the impact of the expansion of the scale of public emergencies on the emergency logistics response capability shows the phenomenon of first enhancement and then gradual decline, as shown in Figure 2.

**Table 9:** Examination of dynamic changes in China’s emergency logistics response capability under the impact of public emergencies.

Brochure	Variant	QR_0.25	QR_0.5	QR_0.75
National sample	Intercept term (C)	−0.3057*** (0.0324)	−0.4220*** (0.0698)	−0.5218*** (0.1035)
	*lnaccident*	0.0507*** (0.00373)	0.06802*** (0.00804)	0.08721*** (0.0119)
Eastern region	Intercept term (C)	−0.626 (−0.6261)	−0.1863 (0.1925)	−0.0194 (0.1423)
	*lnaccident*	0.0885*** (0.0172)	0.050** (0.0218)	0.0399** (0.0161)
Central region	Intercept term (C)	−0.1141*** (0.0865)	0.0447 (0.1463)	−0.1679 (0.1971)
	*lnaccident*	0.0304*** (0.0098)	0.0165 (0.0447)	0.0462** (0.0224)
Western region	Intercept term (C)	−0.225*** (0.0631)	−0.3118*** (0.0805)	−0.468*** (0.1089)
	*lnaccident*	0.040*** (0.00756)	0.0526*** (0.0096)	0.0776*** (0.0130)

*** is significant at the 1% level; ** is significant at the 5% level; * is significant at the 10% level. Same below.

**Figure 3:**
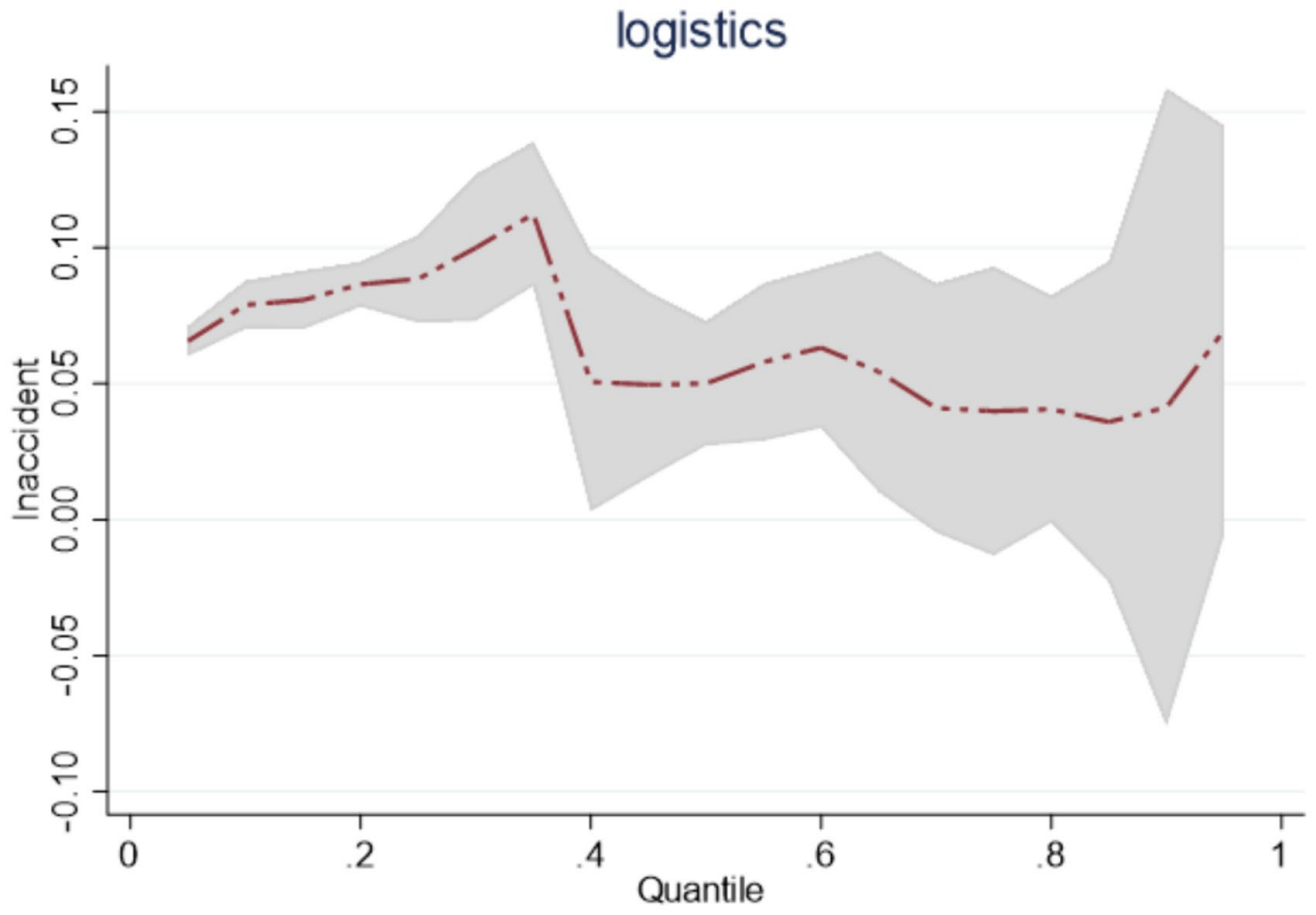
Dynamics of emergency logistics response capability in the eastern region under the impact of public emergencies.

**Figure 4:**
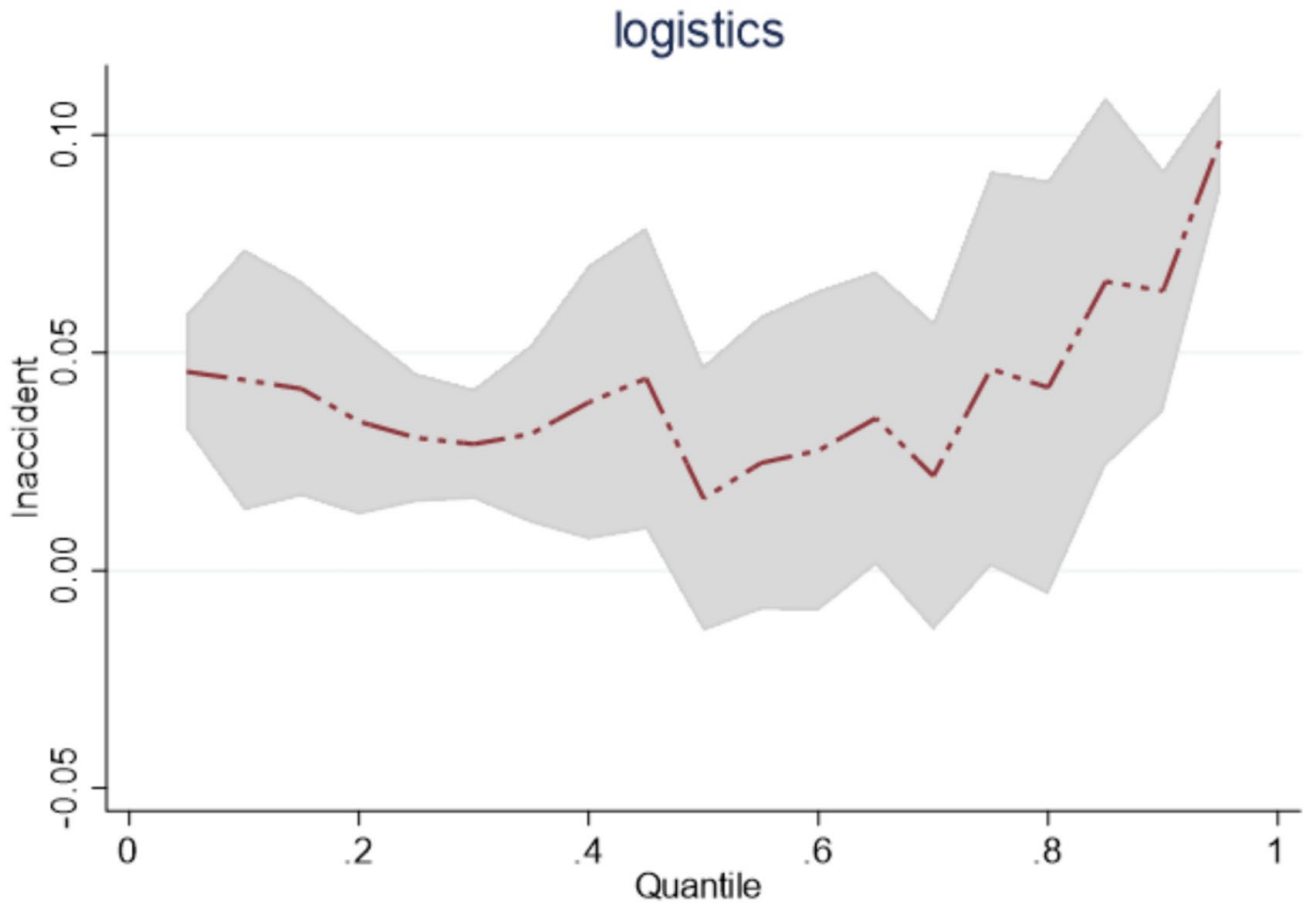
Dynamics of emergency logistics response capability in the central region under the impact of public emergencies.

**Figure 5:**
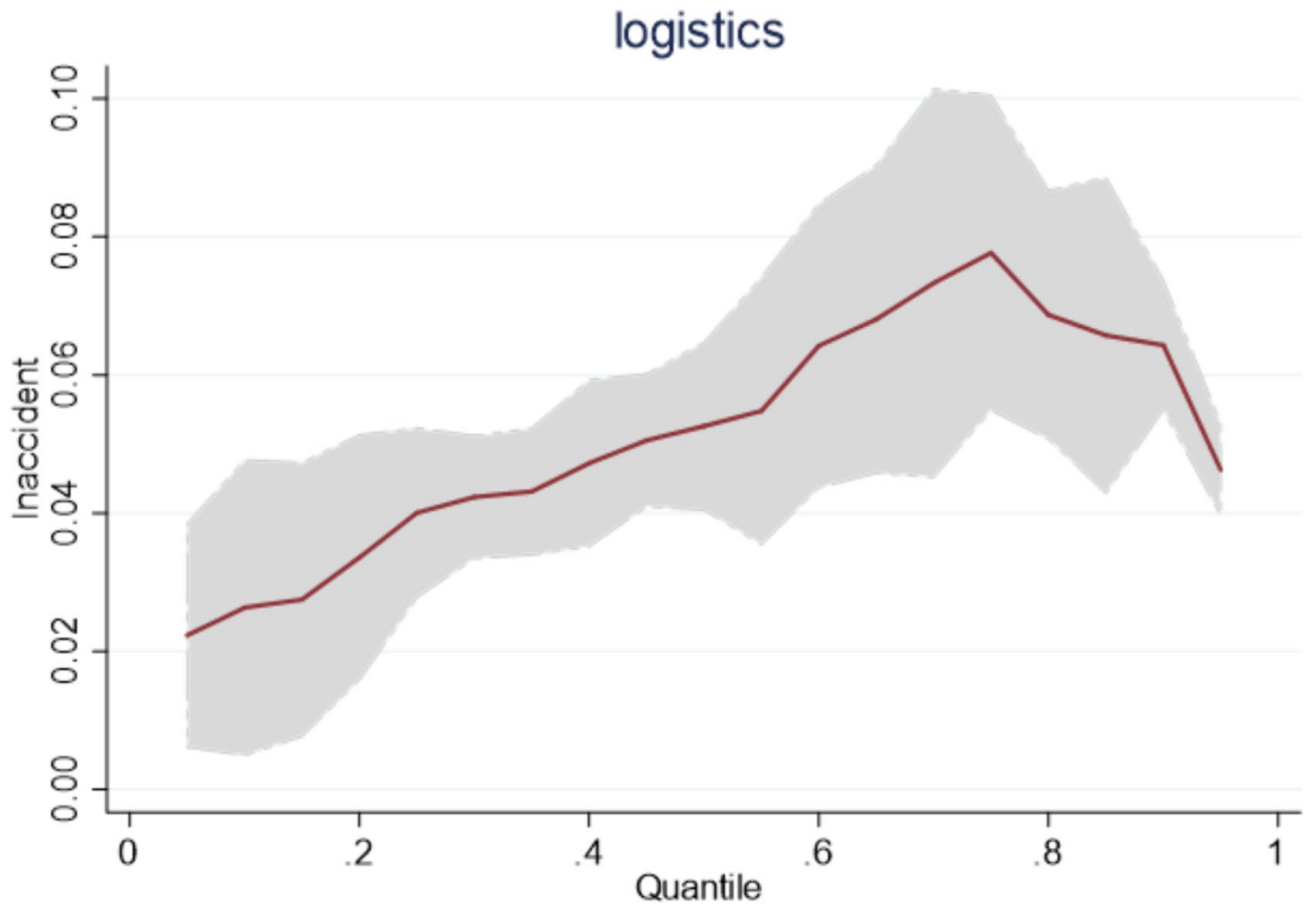
Dynamic changes in emergency logistics response capability in western region under the impact of public emergencies.

Observed by region, the regression coefficients of *lnaccident* at the quantile points of 0.25, 0.5, and 0.75 in the eastern, central, and western regions of China are all significant and positive, but the trends of changes are different in the three regions. Among them, the value of the estimated coefficient of *lnaccident* in the eastern region at the quantile points of 0.25, 0.5, 0.75 is decreasing from 0.0885 to 0.0399. Overall, it shows that as the scale of public emergencies gradually expands, the emergency logistics response capability in the east continues to be in a downward trend, as shown in Figure 3; Central region *lnaccident* in the quartiles 0.25, 0.5, 0.75 of the estimated coefficients of 0.0304, 0.0165, 0.0462. This shows that with the expansion of the scale of public emergencies, the central region of the dynamic changes in the emergency logistics response capability for the first reduction and then enhancement, as shown in Figure 4; Western region *lnaccident* in the quantile points 0.25, 0.5, 0.75 of the estimated coefficients of 0.040, 0.0526, 0.0776. This shows that with the continuous expansion of the scale of public emergencies, the western region emergency logistics response capability continues to improve, as shown in Figure 5.

Reflected from the above regression results. Under the impact of the ever-expanding scale of public emergencies. Western China’s emergency logistics response capabilities continue to improve. And higher than that in the eastern and central regions. The reasons for this phenomenon may be twofold. On the one hand, the scale of public emergencies in the east and central regions is much larger than that in the west, and it is on an upward trend. It may be approaching the capability limit of the emergency management department in the east and central region. On the other hand, due to limited logistics resources, social logistics cannot be completely transformed into emergency logistics. As a result, emergency logistics response capabilities cannot be further improved.

#### 4.3.2 Dynamics of emergency logistics response capability under categorical shocks of public emergencies

Based on the model (4-2), the quantile regression was conducted for the national sample, the eastern region, the central region, and the western region samples. The regression results are shown in Table 10. Observing the regression results of the national sample, the estimated coefficient of *lngeology* at quantiles 0.25, 0.5, and 0.75 grows from −0.002 to −0.020. It is not significant at the low quantile level of 0.25, which indicates that social logistic resources reach their limit as the scale of geologic hazards expands and crosses to the lower scale stage. China’s emergency management department finds it difficult to effectively transform social logistics into emergency logistics, resulting in the failure of emergency logistics response capability. The gradually increasing scale of geologic hazards inhibits the emergency logistics response capability, reflecting the fragile capability of China’s emergency management departments in terms of geologic hazards. *Lnfire*’s estimated coefficients at the tertiles of 0.25, 0.5, and 0.75 take values that increase from 0.015 to 0.098, and then decrease to 0.0493. This suggests that as the scale of the emergency fires continues to expand and rises to a higher level, the emergency logistics response capability will reduce. From the estimated coefficients of *lnenvironment* at quantile points 0.25, 0.5 and 0.75, the regression coefficient rises from 0.019 to 0.0272, and then falls to 0.0194. It shows that as the scale of environmental emergencies continues to expand, emergency logistics response capability are gradually enhanced. However, when the scale of environmental emergencies expands to a higher level, emergency logistics response capability will continue to decrease.

**Table 10:** Dynamic change test of China’s emergency logistics response capability under the categorized shocks of public emergencies.

Brochure	Variant	QR_0.25	QR_0.5	QR_0.75
National sample	Intercept term (C)	0.04** (0.022)	0.149*** (0.027)	0.275*** (0.0312)
	*lngeology*	−0.002 (0.0035)	−0.0135*** (0.004)	−0.02*** (0.0049)
	*lnfire*	0.015*** (0.00514)	0.098** (0.006)	0.0493** (0.00709)
	*lnenvironment*	0.019*** (0.0071)	0.0272*** (0.0087)	0.0194** (0.0097)
Eastern region	Intercept term (C)	0.140*** (0.0315)	0.2219*** (0.0253)	0.3049*** (0.0208)
	*lngeology*	0.00009 (0.0000776)	0.00001 (0.000062)	−0.0000122 (0.000051)
	*lnfire*	−0.000145 (0.000398)	0.00065** (0.000320)	0.000611** (0.000263)
	*lnenvironment*	0.000396 (0.000719)	0.000074 (0.000579)	−0.000256 (0.000476)
Central region	Intercept term (C)	0.1369*** (0.00833)	0.17242*** (0.01353)	0.21053*** (0.0158)
	*lngeology*	−0.0000133** (0.000068)	−0.0000273*** (0.0000108)	−0.0000246** (0.0000127)
	*lnfire*	−0.0000204 (0.0000471)	0.0000502 (0.0000764)	0.0000646 (0.0000892)
	*lnenvironment*	0.003566*** (0.000833)	0.00366*** (0.001352)	0.003425** (0.001579)
Western region	Intercept term (C)	0.0730*** (0.00888)	0.1025*** (0.0103)	0.1095*** (0.01128)
	*lngeology*	0.0000258 (0.0000104)	−0.000048 (0.0000122)	−0.0000293 (0.0000133)
	*lnfire*	0.0002194*** (0.0000665)	0.0004016*** (0.0000778)	0.000565*** (0.0000844)
	*lnenvironment*	0.000596* (0.000360)	0.000244 (0.000422)	0.000390 (0.0004583)

## 5 Paths for improving China’s regional emergency logistics response capability

### 5.1 Indicator selection and data sources

The study findings indicate that the emergency logistics response capability is greater in the eastern region of China compared to the western region, while the central region has the lowest. The emergency logistics response capabilities in the eastern, central, and western areas exhibited a declining trend. Furthermore, it was noted that when the magnitude of public events escalated, China’s capability for emergency logistics response initially grew and then declined. This indicates that when the magnitude of public catastrophes grows, the ability to respond effectively in terms of logistics diminishes. The majority of the regional observations likewise exhibit this comparable pattern. By thoroughly examining and evaluating both local and foreign literature, this paper has condensed the precise aspects that impact the capability of emergency logistics response. This paper further investigates methods to improve China’s capabilities for emergency logistics response. The selection of individual variables is determined in the following manner.

#### 5.1.1 Dependent variable

Emergency logistics response capability (*logistic*): This paper takes emergency logistics response capability as the dependent variable. The data obtained by combining the above calculation methods for emergency logistics response capabilities are used as alternative indicators. The specified observation period is from 2012 to 2021.

#### 5.1.2 Independent variables

Logistics industry fixed asset investment (*K*): Investment in fixed assets in the logistics industry is mainly concentrated in the field of transportation infrastructure construction, including railways, ships, and aircraft. This encompasses the establishment of evacuation railways for ports and logistics hubs, railway logistics centers, and nationwide transportation and logistics public information platforms. These investment sectors are crucial for developing logistical infrastructure that is necessary for improving the capability of emergency logistics response. Nevertheless, due to the time required for infrastructure development, the impact of fixed asset investment on emergency logistics response capability in the short term may not be instantaneous, but rather have a delayed effect (32). Therefore, this paper uses the total fixed asset investment in the logistics industry as an alternative variable based on the research of Malhotra and Mishra (33). In addition, in order to further examine the lag effect of fixed capital investment on emergency logistics response capability, this paper uses the lag term of fixed capital investment as an alternative variable based on Hou (34) and Wang (35).

Logistics industry labor input (*L*): The input of labor significantly influences the growth of the logistics industry, and the labor input in the logistics industry plays a crucial role in improving the capability of emergency logistics response. Therefore, increasing the amount of labor input leads to a greater integration of the labor force with emergency logistics equipment for rescue and protection. This integration effectively enhances the capability of emergency logistics response. Since the logistics industry is composed of traffic, transportation, warehousing, and postal industries, based on the availability of data, this paper draws on the method of Liang (36) and uses the number of workers in the traffic, transportation, warehousing, and postal industries as alternative variables.

Government Expenditures (*Gov*): Government expenditures primarily encompass cash outlays for public services and goods delivered by the government. The government ensures the smooth functioning of society through market-oriented procurement and direct provision (37). Emergency logistical assistance is a component of the public services provided by society. Increasing the government’s financial expenditures directly contributes to improving the emergency logistics response capability. Thus enhancing emergency logistics response capabilities. According to Ma and Guo (38), this paper uses the proportion of provincial government public fiscal expenditure to regional GDP as a proxy variable to indicate the intensity of government fiscal expenditure.

Regional openness (*Open*): Increased regional openness facilitates the adoption of increasingly sophisticated technology and managerial expertise. By incorporating these sophisticated technology and management practices, the regional logistics sector may greatly enhance its capability to respond to emergency logistics situations (39). Moreover, locations that exhibit a high degree of openness typically possess a more advanced import and export commerce. The growth of import and export commerce necessitates enhanced logistics development, hence driving the enhancement of regional logistics efficiency. During public emergencies, social logistics may be rapidly converted into emergency logistics to promote efficiency and hence strengthen the response capabilities of emergency logistics. According to Zhang et al. (40), this paper uses the ratio of total imports and exports to regional GDP as an alternative indicator to measure regional openness.

Regional economic development level (*GDP*): Since the logistics industry is a derivative industry, it is closely related to the level of regional economic development. The higher the level of economic development in a region, the higher the requirements for logistics development (41). Simultaneously, the rise in logistics advancement enables a more efficient conversion of social logistics into emergency logistics when an unforeseen public incident arises, hence enhancing the capability for emergency logistics response. According to Fernald and Li (42), this paper uses gross social product as an indicator to measure the level of regional economic development.

Logistics development level (*logisticGDP*): A region’s greater logistics development level signifies the synchronized growth of the logistics market, robust service capabilities, and comparatively elevated service standards of multi-tier logistics firms (43). Furthermore, the infrastructure plan that supports the growth of logistics is also more rational, resulting in a smoother link between logistics and transportation, and a more sufficient service capability of the comprehensive transportation system. These elements can significantly improve the capability of emergency logistics response. According to Park and Park (44), this paper uses the added value of the logistics industry as an indicator of the degree of logistics development. The definitions and descriptions of specific variables can be found in Table 11.

**Table 11:** Definitions and descriptions of variables.

Variable type	Variable name	Variable symbol	Variable definition
Implicit variable	Emergency logistics response capability	logistic	According to the entropy weight TOPSIS method of this paper to measure the value of the
Independent variable	Investment in fixed assets in the logistics industry	k	Total fixed asset investment in logistics industry
	Labor inputs to the logistics industry	L	Number of employees in the transportation, warehousing and postal industries
	Government expenditure	gov	Government fiscal expenditure/regional GDP
	Openness	open	Total exports and imports/regional GDP
	Level of regional economic development	gdp	Gross social product (GSP)
	Level of logistics development	logisticgdp	Value added of the logistics industry

### 5.2 Model construction

Considering the research goals of this work and the chosen dependent and independent variables in the study mentioned above. In order to test the direct impact of variables such as logistics fixed asset investment (*K*), logistics labor input (*L*), government expenditure (*Gov*), regional openness (*Open*), regional economic development level (*gdp*), and logistics development level (*logisticsgdp*) on emergency logistics response capabilities, this part of the study uses panel data regression methods to test the path to improve emergency logistics response capabilities. This paper selects a panel regression model based on the characteristics of the research data, and the specific form is as follows:


(5-1) $$\begin{array}{l} lnlogistic_{it}=\alpha_{1}+\beta_{1}lnk_{it}+\beta_{2}lnL_{it}+\beta_{3}lngov_{it}\\ +\beta_{4}lnopen_{it}+\beta_{5}lngdp_{it}+\beta_{6}lnlogsiticgdp_{it}+\delta_{it} \end{array}$$

In order to further test the moderating effect of regional openness (*Open*) on regional economic development level (*gdp*) and logistics development level (*logisticsgdp*), and the moderating effect of regional economic development level (*gdp*) on logistics development level (*logisticsgdp*), this section constructs a panel regression model containing the moderating effect as follows:


(5-2) $$\begin{array}{l} lnlogistic_{it}=\alpha_{1}+\beta_{1}lnk_{it}+\beta_{2}lnL_{it}\\ +\beta_{3}lngov_{it}+\beta_{4}lnopen_{it}+\beta_{5}lngdp_{it}\\ +\beta_{6}lnlogsiticgdp_{it}+\beta_{7}lnopen_{it}\times lngdp_{it}\\ +\beta_{7}lnopen_{it}\times lnlogsiticgdp_{it}\\ +\beta_{8}lngdp_{it}\times lnlogsiticgdp_{it}+\delta_{it} \end{array}$$

In addition, in order to further examine the lag effect of fixed asset investment (K) in the logistics industry on emergency logistics response capabilities. This part of the construction contains the $\ln k_{t-n}$ term with n times of time lag. The amount of lags required for investment in fixed assets in the logistics sector to have a positive influence on emergency logistics response capability varies on the specific lagged term. The panel model with lag term is constructed as follows:


(5-3) $$\begin{array}{l} lnlogistic_{it}=\alpha_{1}+\beta_{1}lnk_{it}+\beta_{2}lnL_{it}\\ +\beta_{3}lngov_{it}+\beta_{4}lnopen_{it}+\beta_{5}lngdp_{it}\\ +\beta_{6}lnlogsiticgdp_{it}+\beta_{7}lnopen_{it}\times lngdp_{it}\\ +\beta_{7}lnopen_{it}\times lnlogsiticgdp_{it}+\beta_{8}lngdp_{it}\\ \times lnlogsiticgdp_{it}+\beta_{8+n}lnk_{it-n}\cdots +\delta_{it} \end{array}$$

In the above formula, $lnlogistic_{it}$ represents the emergency logistics response capability; $\ln k_{it}$ represents the fixed asset investment in logistics industry; $\;\ln L_{it}$ represents the labor input in logistics industry; $lngov_{it}$ represents the government expenditure; $lnopen_{it}$ represents the degree of regional openness; $lngdp_{it}$ represents the level of regional economic development; $lnlogisticgdp_{it}$ represents the regional logistics development level. $lnopen_{it}\times lngdp_{it}$ , $lnopen_{it}\times lnlogisticgdp_{it}$ respectively represent the regulatory effect of regional openness on regional economic development level and logistics development level. $lngdp_{it}\times lnlogisticgdp_{it}$ represents the moderating effect of regional economic development level on logistics development level. *it* represents different provinces (municipalities directly under the central government and autonomous regions) in China from 2012 to 2021. $\ln k_{it-n}\;$ represents the number of lagged period of fixed asset investment in logistics industry.

### 5.3 Analysis of regression results

#### 5.3.1 National sample regression results

In the national sample regression analysis of model 5-1, (presented in Table 12), the regression coefficient for the main effect of *lnlogsiticgdp* is not statistically significant. This implies that the extent of logistics development has a restricted influence on the capability of emergency logistics response, maybe due to the inadequate efficiency and overall level of logistics development in China (45). In addition, the regional uneven characteristics of logistics development in China may be more obvious due to the uneven regional development in China. Cross-regional emergency logistics activities need to achieve regional coordination, but regional non-equilibrium characteristics cannot effectively achieve cross-regional emergency logistics coordination. The primary regression coefficient for *lnk* is −0.0177, which is statistically significant. This suggests that an increase in fixed asset investment in the logistics business may hinder the short-term capability for emergency logistics response. The primary regression coefficient for *lnl* is 0.0303, which is statistically significant. This suggests that increasing labor input in the logistics business can improve the capability of emergency logistics response. The regression coefficient for *lngov* is 0.0875, which is statistically significant. This suggests that an increase in government financial expenditure is beneficial for improving the emergency logistical response capabilities. The regression coefficient for the main effect of *lnopen* is 0.0212, which is statistically significant. This suggests that an increase in regional openness can effectively improve the capability for emergency logistics response. The main reason may be that the higher the level of regional openness, the more obvious the effect of attracting foreign investment. In particular, attracting more foreign investment in emergency logistics technology will help to exert the positive spillover effect of advanced emergency logistics technology and management experience on my country’s emergency logistics activities. The primary regression coefficient for *lngdp* is 0.12702, which is statistically significant. This suggests that an increase in the degree of economic development has a favorable impact on the enhancement of emergency logistical response capability. At the same time, it also implies that the higher the level of regional economic development, the higher the demand for high-quality logistics. To improve the quality of logistics development, it is necessary to continue to optimize logistics infrastructure construction, improve logistics transportation tools, improve the quality of the logistics industry labor force, and improve the level of logistics digitalization. The above comprehensive improvement of logistics operation efficiency will indirectly promote the improvement of emergency logistics response capabilities. Therefore, economic development must promote the improvement of emergency logistics response capabilities. Correspondingly, it is necessary to continuously optimize logistics infrastructure, continuously improve logistics transportation tools, enhance the quality of the workforce, increase investment in digitalization of the logistics industry, and improve logistics operation efficiency. The regression coefficients of the above variables show that for the national sample, the impact of economic growth on emergency logistics response is more significant than the impact of government fiscal expenditure, labor input in the logistics industry, and the degree of regional openness to the outside world.

**Table 12:** Path test for improving China’s emergency logistics response capability (national sample).

Variant	Models (5-1)	Models (5-2)	Models (5-3)
Intercept term (C)	−1.0768*** (0.1091)	4.1461*** (0.5849)	−1.185*** (0.14538)
*lnlogisticgdp*	−0.0111 (0.00859)	−0.3369*** (0.0791)	−0.01328 (0.00868)
*lnk*	−0.0177** (0.00722)	−0.0243*** (0.00597)	−0.00037 (0.01490)
*lnl*	0.0303** (0.01588)	0.0214* (0.01294)	0.0178 (0.02183)
*lngov*	0.0875*** (0.0154)	0.0827*** (0.0126)	0.10751*** (0.0252)
*lnopen*	0.0212*** (0.00579)	−0.2085*** (0.0482)	0.00497 (0.00844)
*lngdp*	0.12702*** (0.0162)	−0.4876*** (0.0803)	0.1314*** (0.0219)
lnopen*lngdp		0.0347*** (0.0086)	
lnopen*lnlogisticgdp		−0.0180*** (0.00801)	
lngdp*lnlogisticgdp		0.0478*** (0.00475)	
$lnk_{t-1}$			0.00437 (0.01444)
$lnk_{t-2}$			−0.00832 (0.01449)
$lnk_{t-3}$			0.0201* (0.01165)
F-statistic	52.49***	65.69***	17.53***
R^2^	0.7087	0.6123	0.6893
Regression method	FE	FE	FE

*** is significant at the 1% level; ** is significant at the 5% level; * is significant at the 10% level.

The regression coefficients for the interactions lnopen*lngdp, lnopen*lnlogisticgdp, and lngdp*lnlogisticgdp were obtained from model 5-2. The coefficient for lnopen*lngdp is 0.0347 and statistically significant. This suggests that the moderating effect of *lnopen* on *lngdp* is both significant and positive. This suggests that a higher level of openness to international trade contributes to strengthening the impact of economic growth on the ability to respond to emergency logistics. Conversely, the regression coefficient for the variable lnopen*lnlogsiticgdp is −0.0180 and statistically significant. This suggests that as China becomes more exposed to the global market, the impact of its logistics development level on improving emergency logistics response capabilities diminishes. This reflects the weak competitiveness of China’s logistics industry development. As the degree of opening up to the outside world gradually deepens, with the continuous introduction of foreign logistics companies, Chinese logistics companies need help to compete effectively with foreign logistics companies. Moreover, Chinese logistics companies may be gradually eliminated by the market or gradually lose market initiative in the competition, which is not conducive to improving China’s emergency logistics response capabilities. The regression coefficient for the interaction term lngdp*lnlogisticgdp is 0.0478, and it is statistically significant. This suggests that the variable *lngdp* has a large and positive impact on the variable *lnlogisticgdp*. It also suggests that increasing the level of economic development can contribute to improving the level of logistics development, hence enhancing the capability of emergency logistics response.

In order to further examine the lag effect of fixed asset investment in the logistics industry on emergency logistics response capabilities, a regression was conducted based on model 5-3. The regression analysis reveals that the improvement effect of $\ln k_{t-3}$ on emergency logistics response capability is significant only after *lnk* lags for three periods. Additionally, it also reflects that it takes an average of three years after fixed asset investment in China’s logistics industry to have a positive effect on improving emergency logistics response capabilities. The main reason is that logistics and transportation infrastructure require a certain period from construction to completion and use. However, investments in fixed assets such as logistics infrastructure can only improve emergency logistics response capabilities when they are completed and used.

#### 5.3.2 Sub-regional sample regression

Regression analysis was performed on the sample from the eastern area using model 5-1. The outcomes are presented in Table 13. The regression coefficient for the main effect of *lnlogisticgdp* is statistically insignificant, suggesting that the degree of development in the logistics business in the eastern area remains low. The regression coefficient for the variable *lnk* is 0.1283, indicating a substantial main impact. However, the three-period lag term for this variable is not statistically significant. This suggests that the creation of logistics and transportation infrastructure in the eastern area is highly effective, and the implementation of *lnk* promptly and significantly enhances the potential for emergency logistics response. Furthermore, the primary regression coefficient of *lnl* is 0.0117 and statistically significant. This suggests that the labor input in the logistics industry plays a role in improving the emergency logistics response capability in the eastern region. The regression coefficient for *lngov* is 0.0564 and statistically significant. This suggests that a rise in the government’s financial expenditures in the eastern area is beneficial for improving the emergency logistical response capability. Conversely, the regression coefficient for the variable *lnopen* is 0.1197 and statistically significant. This suggests that a growth in openness in the eastern area contributes to enhancing the capability for emergency logistical response. The regression coefficient for *lngdp* is 0.1719, demonstrating a strong beneficial influence of economic growth in the eastern area on the promotion of emergency logistics response capabilities.

**Table 13:** Path test for the improvement of emergency logistics response capability in the eastern region.

Variant	Models (5-1)	Models (5-2)	Models (5-3)
Intercept term (C)	−3.7157*** (0.4568)	0.0299 (2.580)	−2.448*** (0.6526)
*lnlogisticgdp*	−0.0163 (0.0263)	−0.1325 (0.4164)	0 0.0098 (0.0305)
*lnk*	0.1283*** (0.0310)	0.0695* (0.0338)	0.1289** (0.0560)
*lnl*	0.0117*** (0.0342)	0.0040 (0.0321)	0.0010 (0.04132)
*lngov*	0.0564* (0.0338)	0.0660*** (0.03146)	0.0795* (0.0478)
*lnopen*	0.1197*** (0.0238)	0.03621 (0.214)	−0.0242 (0.04333)
*lngdp*	0.1719*** (0.0389)	−0.2648 (0.3995)	0.0598 (0.0541)
lnopen*lngdp		0.0217 (0.0421)	
lnopen*lnlogisticgdp		−0.0254 (0.0434)	
lngdp*lnlogisticgdp		0.03492*** (0.01242)	
$lnk_{t-1}$			−0.0089 (0.0760)
$lnk_{t-2}$			0.0410 (0.1034)
$lnk_{t-3}$			0.0785*** (0.0590)
F-statistic	42.38***	34.51***	20.15***
R^2^	0.6962	0.7826	0.5527
Regression method	FE	FE	FE

*** is significant at the 1% level; ** is significant at the 5% level; * is significant at the 10% level.

The regression coefficients for the interaction terms lnopen*lngdp, lnopen*lnlogisticgdp, and lngdp*lnlogisticgdp in the eastern area are obtained using model 5-2. The regression coefficient for the interaction term lngdp*lnlogisticgdp is 0.03492, and it is statistically significant. This suggests that the variable *lngdp* has a significant and positive impact on *lnlogisticgdp* in the eastern region. In other words, as the level of economic development increases in the eastern region, it leads to an improvement in the level of logistics development and enhances the capability of emergency logistics response. Neither the regression coefficient for lnopen*lngdp nor the regression coefficient for lnopen*lnlogisticgdp are statistically significant. This suggests that the amount of openness in the eastern area may already be rather high, and increasing the degree of openness to the outside world cannot further improve the function of economic development in enhancing emergency logistics response capability. Hence, it is imperative to strengthen the quality of openness to external influences as the primary objective for improving the degree of openness in the future.

The lag effect of model 5-3 logistics industry fixed asset investment on the emergency logistics response capability in the eastern region was observed. The improvement effect of $lnk_{t-3}$ on the emergency logistics response capability was significant only after *lnk* lags for three periods. It shows that investment in fixed assets of logistics infrastructure in the eastern region can improve emergency logistics response capabilities only after three years.

The regression analysis for the central area sample in model 5-1 (refer to Table 14) indicates that the regression coefficient for the main effect of *lnlogisticgdp* is 0.0227. This suggests that the degree of logistics advancement in the central area positively contributes to improving the ability to respond to emergency logistics. The primary factor behind this is the central region’s strategic location, which serves as a connecting point between the east and west, as well as the north and south. Furthermore, the focal point of China’s logistics industry development has shifted from the eastern region to the central part of the country (46). Conversely, the primary regression coefficient for *lnk* is −0.0305 and statistically significant, but the primary regression coefficient for *lnl* is −0.0137 but lacks statistical significance. This shows that labor input in the logistics industry in the central region still needs to be increased. Although the development focus of China’s logistics industry has shifted to the central region, labor input in this region may still need to be able to effectively support the development of the logistics industry, which shows that labor input does not significantly improve emergency logistics response capabilities. Therefore, the central region should increase labor investment in the logistics industry and attract more labor forces to enter the logistics industry. Furthermore, the regression coefficient of *lngov* is 0.0893 and statistically significant. This suggests that a growth in governmental financial expenditures contributes to the enhancement of emergency logistical response capability in the central area. Therefore, the central region government should further increase fiscal expenditures on the logistics industry, effectively promote the construction of public logistics infrastructure in the central region, and increase financial subsidies for developing the logistics industry. The regression coefficient for the main effect of *lnopen* is 0.0893 and statistically significant. This suggests that a rise in openness in the center area contributes to the enhancement of emergency logistical response capability. The central region still needs to be opened compared with the eastern region of China. According to historical data, the proportion of foreign investment attracted by the eastern region continues to be as high as 80%, while the central region accounts for a lower proportion. Therefore, the central region should increase regional opening up, increase its attractiveness to foreign investment, and enhance the positive spillover effect of foreign investment on the region’s economic and logistics development. The regression coefficient for the variable *lngdp* is 0.0999, showing a substantial positive effect. This suggests that an increase in economic growth in the central area is beneficial for improving emergency logistical response capability.

**Table 14:** Path test for the improvement of emergency logistics response capability in the central region.

Variant	Models (5-1)	Models (5-2)	Models (5-3)
Intercept term (C)	−0.6578*** (0.1032)	1.639 (2.0363)	−0.6751*** (0.159)
*lnlogsiticgdp*	0.0227** (0.013)	−0.1910 (0.2542)	−0.000258 (0.01591)
*lnk*	−0.0305*** (0.0044)	−0.0344*** (0.0050)	−0.0318*** (0.01024)
*lnl*	−0.0137 (0.01782)	−0.0113 (0.0173)	0.0289 (0.0467)
*lngov*	0.0893*** (0.0133)	0.0811*** (0.0131)	0.05987* (0.0323)
*lnopen*	0.03019*** (0.00786)	−0.0072 (0.2059)	0.0168 (0.01434)
*lngdp*	0.0999*** (0.0158)	−0.1716 (0.2883)	0.0898*** (0.0289)
lnopen*lngdp		0.0108 (0.0349)	
lnopen*lnlogsiticgdp		−0.0112 (0.0307)	
lngdp*lnlogsiticgdp		0.0282** (0.0111)	
$lnk_{t-1}$			0.000158 (0.00913)
$lnk_{t-2}$			−0.00976 (0.00893)
$lnk_{t-3}$			0.01374** (0.0073)
F-statistic	45.51	33.90	11.9
R^2^	0.5140	0.4976	0.5272
Regression method	FE	FE	FE

*** is significant at the 1% level; ** is significant at the 5% level; * is significant at the 10% level.

The regression analysis of model 5-2 reveals that the only significant regression coefficient among the moderating effects of lnopen*lngdp, lnopen*lnlogisticgdp, and lngdp*lnlogisticgdp in the central area is 0.0282. This suggests that when the degree of economic growth in the central area rises, it enhances the impact of logistics development on the capability to respond to emergencies in the field of logistics. The regression coefficients for lnopen*lngdp and lnopen*lnlogisticgdp are not statistically significant. But the contrast with the eastern area is attributable to the lesser degree of opening up of the central region to the outside world.

From the lag effect of model 5-3 logistics industry fixed asset investment on emergency logistics response capabilities, it can be seen that the improvement effect of $lnk_{t-3}$ on emergency logistics response capabilities is significant only after *lnk* lags for three periods. It shows that investment in fixed assets of logistics infrastructure in the central region can improve emergency logistics response capabilities only after three years. However, the impact in the central region is lower than in the eastern region. Therefore, the eastern region must further improve the investment efficiency of fixed assets in the logistics industry.

The regression analysis of model 5-1, using a sample from the western area, reveals that the regression coefficient for the main effect of *lnlogisticgdp* is not statistically significant, as indicated in Table 15. The primary factor behind this is the comparatively limited advancement of the logistics sector in the western area. Due to the uneven development of China’s logistics industry, the eastern region is larger than the central region, and the development level of the western region is the weakest. Therefore, the western region must improve the level of logistics development, accelerate the transformation and optimization of logistics infrastructure, improve the digitalization level of the logistics industry, optimize logistics transportation tools, and thereby improve the efficiency of logistics operations. Furthermore, the regression coefficients for both *lnk* and *lnl* are similarly statistically insignificant. The reason for this may be because the logistics business in the western area follows an outdated development strategy, which primarily depends on increasing the size of the workforce (46). However, this strategy is no longer viable. In addition, the logistics industry’s development model in the western region faces a serious “dual attribute” contradiction. On the one hand, it drives logistics development through the expansion of capital scale, and on the other hand, it relies on improving labor efficiency. This development model with dual attributes has an inhibitory effect on the industry’s development (47). The regression coefficient for *lngov* is 0.0662, which is statistically significant. This suggests that increasing government financial investment in the western area has a positive impact on improving emergency logistical response capability. Therefore, the western region should be treated like the central region. The government of the western region should further increase fiscal expenditures on the logistics industry, effectively promote the construction of public logistics infrastructure in the central region, and increase financial subsidies for its development. Furthermore, the primary regression coefficient of *lnopen* is 0.01315 and statistically significant. This suggests that a growth in the degree of openness in the western area contributes to the enhancement of emergency logistical response capability. Finally, the main effect regression coefficient of *lngdp* is 0.0945 and significant, showing that the increase of economic development level in the western area is beneficial to enhance the emergency logistical response capability.

**Table 15:** Path test for the improvement of emergency logistics response capability in the western region.

Variant	Models (5-1)	Models (5-2)	Models (5-3)
Intercept term (C)	−0.6274*** (0.0717)	1.4058*** (0.4713)	−0.37539** (0.1586)
*lnlogsiticgdp*	0.00141 (0.00489)	−0.2385*** (0.0658)	0.00106 (0.0050)
*lnk*	−0.00855 (0.00682)	−0.00878 (0.00595)	−0.0282 (0.0172)
*lnl*	−0.0108 (0.0120)	−0.0052 (0.01056)	0.0122 (0.017)
*lngov*	0.0662*** (0.0175)	0.07284*** (0.0156)	0.0257 (0.0336)
*lnopen*	0.01315*** (0.0033)	−0.0815** (0.0333)	0.00976 (0.0066)
*lngdp*	0.0945*** (0.0121)	−0.0952 (0.06724)	0.0300 (0.0339)
lnopen*lngdp		0.0060 (0.0070)	
lnopen*lnlogsiticgdp		0.00593 (0.00661)	
lngdp*lnlogsiticgdp		0.0206*** (0.00507)	
$lnk_{t-1}$			0.0046 (0.0105)
$lnk_{t-2}$			0.00352 (0.0109)
$lnk_{t-3}$			0.00172 (0.0121)
$lnk_{t-4}$			0.0301* (0.0186)
F-statistic	31.29***	30.77***	5.71***
R^2^	0.6477	0.4382	0.6528
Regression method	FE	FE	FE

*** is significant at the 1% level; ** is significant at the 5% level; * is significant at the 10% level.

The lag effect of model 5-3 logistics industry fixed asset investment on emergency logistics response capabilities shows that $lnk_{t-4}$ can have a favorable driving effect on emergency logistics response capabilities only after *lnk* lags for four periods. It also shows that the operating efficiency of fixed asset investment in the logistics industry in the western region is lower than that in the eastern and central regions. Therefore, the western region should accelerate the improvement of fixed capital investment efficiency.

## 6 Discussion

This paper explores the dynamic changes in China’s regional emergency logistics response capabilities under the impact of public emergencies and its improvement paths. Firstly, this paper expands the research on emergency logistics capability evaluation and overcomes the existing problems of single indicators and subjective measurement methods in emergency logistics capability evaluation. This study constructs a comprehensive indicator system of emergency logistics response capability based on the components of this capability. The entropy weight TOPSIS method was used to measure the emergency logistics response capabilities of different regions in China, achieving an objective quantitative study of these capabilities. In addition, existing literature on emergency logistics capabilities often only evaluates a single object, resulting in relatively single selected indicators. However, emergency logistics response capability is comprehensive, and a single indicator cannot fully reflect its overall picture. To this end, this paper constructs a multi-dimensional comprehensive indicator system including emergency logistics carrier carrying capability, emergency logistics flow capability, emergency logistics flow rate capability, emergency logistics flow efficiency capability, regional informatization capability, and social emergency guarantee capability. Compared with existing research, this indicator system can more effectively cover the connotation and characteristics of emergency logistics response capabilities and more comprehensively realize the comprehensive evaluation of emergency logistics response capabilities. The findings reveal a significant and rapid enhancement in China’s emergency logistics response capability, with consistent improvements across eastern, central, and western regions. Notably, the eastern region’s response capability exceeds that of the central, which surpasses the western region. From the perspective of regional differences, China’s emergency logistics response capabilities are expanding. The differences in emergency logistics response capabilities of provinces within the eastern region are higher than those in the western region, while the differences within the central region are relatively the lowest.

Secondly, this paper expands the research on public emergencies’ connotations, types, and impacts. Based on the connotation and types of public events, this paper quantifies the dynamic changes in China’s regional emergency logistics response capabilities under the impact of public emergencies and their classification events, enriching the discussion on the impact effects of public emergencies. This study has important implications for exploring the changes in China’s regional emergency logistics response capabilities under the impact of public emergencies and their classification events from an empirical perspective. The study found that: China’s emergency management departments can effectively transform social logistics into emergency logistics and promote the improvement of emergency logistics response capabilities. However, judging from the impact trend, when the scale of public emergencies expands at a high level, China’s emergency management departments cannot further transform social logistics into emergency logistics, resulting in a gradual decline in emergency logistics response capabilities. Regional observation: Under the impact of the ever-expanding scale of public emergencies, the emergency management departments in the western region have continuously improved their emergency logistics response capabilities, which are higher than those in the eastern and central regions. The research on the impact of classified public emergencies found that as the scale of sudden geological disasters expands and exceeds the lower scale, social logistics resources have exceeded the limit. China’s emergency management departments cannot effectively transform social logistics into emergency logistics, failing emergency logistics response capabilities. This also indirectly reflects the relative weakness of China’s emergency management department in dealing with geological disasters. In the process of converting social logistics into emergency logistics to respond to fires, China’s emergency management departments may have limited their ability to convert social logistics into emergency logistics further as the scale of sudden fires expands to a higher level, resulting in a gradual decline in emergency logistics response capabilities. As the scale of sudden environmental incidents expands, the emergency logistics response capability gradually increases. However, when the scale of sudden environmental incidents expands to a higher level, the emergency logistics response capability will continue to decline. Regional observation: In the process of transforming social logistics into emergency logistics to respond to fires, the emergency management departments in the eastern region may exceed the capability limit of the emergency management departments in the eastern region when the scale of sudden fires rises to a higher level, making it impossible to transform social logistics into emergency logistics further. This leads to a gradual decline in the emergency logistics response capability in the eastern region. For the central region, as the scale of sudden geological disasters expands and exceeds the lower scale, it has exceeded the limit of social logistics resources in the central region, so the emergency management department in the region cannot effectively transform social logistics into emergency logistics. This leads to the failure of emergency logistics response capabilities and then shows that as the scale of geological disasters expands, the inhibition of emergency logistics response capabilities is gradually increasing. However, the inhibitory effect gradually decreases when the scale of sudden geological disasters expands to a relatively high level. For the western region, the region’s emergency management department can continuously summarize practical experience or optimize emergency logistics plans to transform social logistics into emergency logistics to respond to fires, thereby continuously improving emergency logistics response capabilities.

In addition, based on the dynamic changes in regional emergency logistics response capabilities under the impact of public emergencies, this paper summarizes the specific factors affecting emergency logistics response capabilities after combing and analyzing domestic and foreign literature. It also explores the path to improve China’s emergency logistics response capabilities from an empirical research perspective. The study found: the impact of China’s logistics development level on emergency logistics response capabilities is not significant. The main reason may be that although China has become a major logistics country in the world, its development efficiency is low, and the overall level is not high. The increase in labor input in the logistics industry, regional openness, economic development level, and government fiscal expenditure will help improve emergency logistics response capabilities. However, the level of economic development has the most significant impact on improving emergency logistics response capabilities. From a regional perspective, only the level of logistics development in the central region has a positive effect on improving emergency logistics response capabilities. This discrepancy may be due to underdevelopment in the logistics industries of the eastern and western regions. The results of fixed asset investment in the logistics industry and its lag items show that it takes three years in the eastern and central regions and four years in the western regions to produce a positive impact. This reflects that the efficiency of fixed asset investment in the logistics industry in the eastern and central regions is relatively high, and the western region is the lowest. Further expanding the scale of labor input in the eastern region’s logistics industry can effectively promote improving emergency logistics response capabilities. However, the central and western regions cannot improve their emergency logistics response capabilities through this channel. The effect of fiscal expenditure on improving emergency logistics response capabilities in the central region is more potent than in the western region. At the same time, it is weakest in the eastern region. The economic development level in the eastern region substantially affects the emergency logistics response capability more than the central and western regions. The eastern region has a positive regulatory effect on logistics development through the economic development level, which enhances the driving effect of the logistics development level on emergency logistics response capability. This effect is higher than that in the central and western regions. Regional observation: Only the logistics development level in the central region promotes improving emergency logistics response capabilities. The results of fixed asset investment in the logistics industry and its lag items show that it takes three years in the eastern and central regions and four years in the western regions to produce a positive impact. This reflects that the efficiency of fixed asset investment in the logistics industry in the eastern and central regions is relatively high, and the western region is the lowest. Further expanding the scale of labor input in the eastern region’s logistics industry can effectively promote improving emergency logistics response capabilities. However, the central and western regions cannot improve their emergency logistics response capabilities through this channel. The effect of fiscal expenditure on improving emergency logistics response capabilities in the central region is more potent than in the western region. At the same time, it is weakest in the eastern region. The economic development level in the eastern region substantially affects the emergency logistics response capability more than the central and western regions. The eastern region has a positive regulatory effect on logistics development through the economic development level, which enhances the driving effect of the logistics development level on emergency logistics response capability. This effect is higher than that in the central and western regions. In addition, the economic development level in the eastern region has a more substantial effect on the emergency logistics response capability than the central and western regions. The eastern region has a positive regulatory effect on logistics development through the economic development level, which enhances the driving effect of the logistics development level on emergency logistics response capability. This effect is higher than that in the central and western regions and weakest in the western region.

In practice, as the world’s second-largest economy, China has the most complex and comprehensive terrain and landforms, and the types of sudden public events are also diverse. Compared with other countries, China’s emergency logistics system has more systematic and comprehensive experience in responding to public emergencies. Moreover, this paper explores the dynamic changes and improvement paths of China’s regional emergency logistics response capabilities under the impact of public emergencies. It will help to clarify whether different regions of China can provide adequate emergency guarantees for the various resources required for international direct investment. It can also provide decision-making references for international direct investment market entities to make scientific regional choices. Precisely, in selecting investment regions in China, This article can accurately judge the impact of public emergencies in different regions on direct investment risks, reduce the adverse impact of public emergencies on international direct investment, and improve the efficiency of international direct investment.

## 7 Conclusion

### 7.1 Full text summary

1. This study employs the entropy weight TOPSIS method to quantitatively evaluate regional emergency logistics response capabilities, addressing the gap in current research that tends towards subjective or purely quantitative analysis without sufficient empirical investigation. The research introduces the “regional informatization capability” indicator, contributing to a more scientifically valid measurement of emergency logistics response speed. The findings reveal a significant and rapid enhancement in China’s emergency logistics response capability, with consistent improvements across eastern, central, and western regions. Notably, the eastern region’s response capability exceeds that of the central, which surpasses the western region. While the eastern and central areas score above the national average in emergency logistics response, the western region lags behind. Additionally, the study observes increasing regional disparities in China, with the eastern provinces exhibiting greater variability in response capabilities compared to the western region, and the central region showing the least variability.
2. This paper explores the dynamic changes in China’s regional emergency logistics response capabilities under the impact of public emergencies and their classification events. This study solves the problem of quantitative research on the types of public emergencies and their impacts and expands the impact effects of public emergencies. Key findings include:

China’s disaster management authorities demonstrate an efficient conversion of social logistics into emergency logistics, strengthening emergency response capability. However, this capability diminishes during extensive emergencies, leading to reduced effectiveness in emergency logistics response. The western region now exceeds the east-central area in emergency response capabilities due to increased public emergency events.

The study identifies a critical limitation in emergency logistics response during large-scale geologic disasters, indicating a vulnerability in China’s emergency management system. Fire crises impact the transformation of social logistics into emergency logistics, weakening emergency response capabilities. Emergency logistics response to environmental disasters shows an initial increase, followed by a decrease as the event’s magnitude grows.

Regionally, the eastern area faces challenges in managing unexpectedly large fires, impacting the utilization of social logistics for emergency response. In the central region, the increase in geologic disasters initially intensifies and then lessens the impact on emergency logistics response. The study also notes a similar pattern for environmental incidents in this region. In contrast, the western region has consistently improved its fire response capabilities through experience and strategic planning.

1. This research empirically examines strategies to bolster China’s regional emergency logistics response. It addresses a gap in existing literature by empirically exploring ways to enhance regional emergency response capabilities. The study yields several key insights:

First of all, China’s logistics development level does not significantly impact emergency logistics response capabilities. The main reason may be that although China has become a major logistics country in the world, its development efficiency is low, and its overall level is not high. Factors such as increased logistics workforce, regional accessibility, economic growth, and government expenditure positively influence emergency response capabilities. Economic growth is found to have the most substantial impact, followed by government spending, labor input in logistics, and regional openness. In addition, fixed asset investment in China’s logistics industry takes an average of three years to have a positive effect on improving emergency logistics response capabilities.

Secondly, from a regional perspective, only the level of logistics development in the central region has a positive effect on improving emergency logistics response capabilities. This discrepancy may be due to underdevelopment in the logistics industries of the eastern and western regions. The results of fixed asset investment in the logistics industry and its lag items show that it takes three years in the eastern and central regions and four years in the western regions to produce a positive impact. This reflects that the efficiency of fixed asset investment in the logistics industry in the eastern and central regions is relatively high, and the western region is the lowest. Further expanding the scale of labor input in the eastern region’s logistics industry can effectively promote improving emergency logistics response capabilities. However, the central and western regions cannot improve their emergency logistics response capabilities through this channel. The effect of fiscal expenditure on improving emergency logistics response capabilities in the central region is more potent than in the western region. At the same time, it is weakest in the eastern region. The economic development level in the eastern region substantially affects the emergency logistics response capability more than the central and western regions. The eastern region has a positive regulatory effect on logistics development through the economic development level, which enhances the driving effect of the logistics development level on emergency logistics response capability. This effect is higher than that in the central and western regions.

### 7.2 Revelations

This study recommends strategies to enhance China’s regional emergency logistics response capabilities:

1. Coordinated Development and Resource Optimization: There’s a need for stronger resource distribution and coordinated development across regions. Key suggestions include creating a unified information platform for emergency logistics across eastern, central, and western areas, establishing data-sharing systems, and ensuring stable operation of the emergency management system. At the same time, the country must continue to increase communication and collaboration among various regions, referencing or introducing advanced experience, methods, and policies to improve regional emergency logistics response capabilities. Jointly build a plan to improve regional emergency logistics response capabilities and an experience-sharing learning mechanism. In this way, the gap in emergency logistics response capabilities between regions can be narrowed, thereby improving the overall emergency logistics response capabilities across the country.
2. Long-term Emergency Response Framework: Establishing standardized and scientific emergency management measures and improving the system is necessary. This system should focus on classified management response measures for various public emergencies and territorial management. It is essential to ensure that emergency management work is carried out orderly. In conjunction with public emergencies and their classified management measures, the emergency management department should develop emergency plans and conduct simulation drills to enhance emergency response capabilities.
3. Logistics Development and Market Coordination: The research underscores the importance of improving the degree of logistics development and service capabilities of the logistics market. This includes enhancing the service levels of logistics firms, developing logistics infrastructure for seamless transportation, attracting investment in advanced emergency logistics technologies, and increasing R&D in this domain. Additionally, boosting government financial expenditures in disaster logistics infrastructure and emergency personnel training is recommended to further strengthen the emergency response framework.

### 7.3 Shortcomings and outlook of this paper

This paper measures China’s regional emergency logistics response capabilities by constructing the components of emergency logistics response capabilities. It explores the dynamic changes and improvement paths of China’s regional emergency logistics response capabilities under the impact of public emergencies. Due to time constraints and difficulty in obtaining some data, the research in this article still has certain deficiencies and limitations, which await further in-depth research. Firstly, in addition to exploring the dynamic changes in China’s emergency logistics response capabilities under the impact of public emergencies, public emergencies have different response levels, and different types of public emergency response levels have different requirements for emergency logistics response capabilities. For example, according to the impact of public emergencies, public emergencies are generally divided into four levels based on factors such as the nature of the event, losses caused, degree of harm, controllability, and scope of impact: particularly major, significant, relatively large, and general. The emergency level of emergency logistics can be divided into four levels: enterprise-level emergency logistics, regional-level emergency logistics, national-level emergency logistics, and international-level emergency logistics. This article cannot further analyze how to measure the relationship between the two. On the one hand, the existing public data needs to define further the indicators for the classification response level of public emergencies. For example, there is no quantitative definition of the amount of losses and the degree of harm caused by public emergencies. Instead, only “particularly major” Qualitative descriptions such as “significant,” “larger,” “general,” etc. How quantifying the above qualitative description requires a breakthrough and expansion of this part of the content in subsequent research. Secondly, in the study of paths to improve emergency logistics response capabilities, there is no further breakdown of the paths to improve the components of emergency logistics response capabilities. The components of emergency logistics response capabilities include emergency logistics carrier capabilities, emergency logistics flow capabilities, emergency logistics flow efficiency capabilities, social emergency support capabilities, etc. Improving the overall response capability of emergency logistics is based on improving individual capabilities. Therefore, improving the development level of emergency logistics components is the next step that needs to be expanded in research. In addition, public emergencies have different impacts on emergency logistics carrier capabilities, emergency logistics flow capabilities, emergency logistics flow efficiency capabilities, and social emergency support capabilities. This study also did not explore the changes in the components of emergency logistics response capabilities under the impact of public emergencies. Due to the considerable length of the paper and limited time, this article did not continue to study this part, which needs to be further expanded in future research.

## Data availability statement

The raw data supporting the conclusions of this article will be made available by the authors, without undue reservation.

## Author contributions

HC: Writing – original draft, Writing – review & editing. YG: Data curation, Formal analysis, Methodology, Writing – original draft. XL: Investigation, Methodology, Project administration, Writing – original draft. XQ: Conceptualization, Data curation, Formal Analysis, Writing – original draft.

## Footnotes

^1^ https://www.stats.gov.cn/

^2^The eastern region includes 11 provinces (cities) including Beijing, Tianjin, Hebei, Liaoning, Shanghai, Jiangsu, Zhejiang, Fujian, Shandong, Guangdong and Hainan; The central region includes 10 provinces (autonomous regions) including Shanxi, Inner Mongolia, Jilin, Heilongjiang, Anhui, Jiangxi, Henan, Hubei, Hunan, and Guangxi; The western region includes nine provinces (autonomous regions): Sichuan, Guizhou, Yunnan, Tibet, Shaanxi, Gansu, Qinghai, Ningxia, and Xinjiang.

## References

[1] Xue L Liu B. New challenges and top-level design of emergency management system. J Natl Acad Admin. (2013) 1:10-14+129. doi: 10.14063/j.cnki.1008-9314.2013.01.015

[2] Uriel R Michael C Paul T. Coping with crises: the management of disasters, riots, and terrorism. Henan: Henan People's Publishing House (2014) 102–3.

[3] Exum NG Betanzo E Schwab KJ Chen TYJ Guikema S Harvey DE. Extreme precipitation, public health emergencies, and safe drinking water in the USA. Curr Environ Health Rep. (2018) 5:305–15. doi: 10.1007/s40572-018-0200-5 29687348

[4] Mlađan D. Cvetković V. (2013). “Classification of emergency situations.” *Thematic Proceedings of International Scientific Conference Archibald Reiss Days*, pp. 275–291.

[5] Hong L. China's natural disasters economic growth in the long run——an analysis of co-integration relationship based on VAR and VEC models. J Beijing Inst Technol Soc Sci. (2018) 20:112–8. doi: 10.15918/j.jbitss1009-3370.2018.5458

[6] Kostova D Cassell CH Redd JT Williams DE SinghT MLD Bunnell RE. Long-distance effects of epidemics: assessing the link between the 2014 West Africa EbolaOutbreak and US exports and employment. Health Econ. (2019) 28:1248–61. doi: 10.1002/hec.3938, PMID: 31464014 PMC6852535

[7] Ceylan RF Ozkan B. The economic effects of epidemics: from SARS and MERS to COVID-19. Res J Adv Human. (2020) 1:21–9. doi: 10.58256/rjah.v1i2.132

[8] Antràs P Redding SJ Rossi-Hansberg E. Globalization and pandemics. Am Econ Rev. (2023) 113:939–81. doi: 10.1257/aer.20201479

[9] Rasouli MR. Intelligent process-aware information systems to support agility in disaster relief operations: a survey of emerging approaches. Int J Prod Res. (2019) 57:1857–72. doi: 10.1080/00207543.2018.1509392

[10] Rodríguez-Espíndola O Chowdhury S Beltagui A Albores P. The potential of emergent disruptive technologies for humanitarian supply chains: the integration of blockchain, artificial intelligence and 3D printing. Int J Prod Res. (2020) 58:4610–30. doi: 10.1080/00207543.2020.1761565

[11] Lu L Luo XC. Emergency transportation problem based on single-valued neutrosophic set. Discret Dyn Nat Soc. (2020) 2020:1–8. doi: 10.1155/2020/4813497

[12] Ghelichi Z Gentili M Mirchandani P. A simulation-based performance evaluation model for decision support on drone location and delivery scheduling. J Humanit Logist Supply Chain Manag. (2024) in press. doi: 10.1108/JHLSCM-04-2023-0036

[13] Jeong KY Hong JD Xie Y. Design of emergency logistics networks, taking efficiency, risk and robustness into consideration. Int J Log Res Appl. (2014) 17:1–22. doi: 10.1080/13675567.2013.833598

[14] Alem D Clark A Moreno A. Stochastic network models for logistics planning in disaster relief. Eur J Oper Res. (2016) 255:187–206. doi: 10.1016/j.ejor.2016.04.041

[15] Manopiniwes W Irohara T. Stochastic optimisation model for integrated decisions on relief supply chains: preparedness for disaster response. Int J Prod Res. (2016) 55:979–96. doi: 10.1080/00207543.2016.1211340

[16] Boonmee C Arimura M Asada T. Facility location optimization model for emergency humanitarian logistics. Int J Disaster Risk Reduct. (2017) 24:485–98. doi: 10.1016/j.ijdrr.2017.01.017

[17] Maharjan R Hanaoka S. A credibility-based multi-objective temporary logistics hub location-allocation model for relief supply and distribution under uncertainty. Socio Econ Plan Sci. (2020) 70:100727. doi: 10.1016/j.seps.2019.07.003

[18] Ransikarbum K Mason SJ. A bi-objective optimisation of post-disaster relief distribution and short-term network restoration using hybrid NSGA-II algorithm. Int J Prod Res. (2021) 60:5769–93. doi: 10.1080/00207543.2021.1970846

[19] Loree N Aros-Vera F. Points of distribution location and inventory management model for post-disaster humanitarian logistics. Transp Res E Logist Transp Rev. (2018) 116:1–24. doi: 10.1016/j.tre.2018.05.003

[20] Yuan Y Wang F. An emergency supplies scheduling for chemical industry park: based on super network theory. Environ Sci Pollut Res. (2022) 29:39345–58. doi: 10.1007/s11356-021-18182-y, PMID: 35099704

[21] Sheu JB. Dynamic relief-demand management for emergency logistics operations under large-scale disasters. Transp Res E Logist Transp Rev. (2010) 46:1–17. doi: 10.1016/j.tre.2009.07.005

[22] Moltchanova E Khabarov N Obersteiner M Ehrlich D Moula M. The value of rapid damage assessment for efficient earthquake response. Saf Sci. (2011) 49:1164–71. doi: 10.1016/j.ssci.2011.03.008

[23] Nachtmann H Pohl EA. Transportation readiness assessment and valuation for emergency logistics. Int J Emerg Manag. (2013) 9:18–36. doi: 10.1504/IJEM.2013.054099

[24] Ma G Tan S Shang S. The evaluation of building fire emergency response capability based on the CMM. Int J Environ Res Public Health. (2019) 16:1962. doi: 10.3390/ijerph16111962, PMID: 31163571 PMC6603904

[25] Jiang Y Yuan Y. Emergency logistics in a large-scale disaster context: achievements and challenges. Int J Environ Res Public Health. (2019) 3:779. doi: 10.3390/ijerph16050779 PMC6427432 30836640

[26] Zhang Y Ding Q Liu J. Performance evaluation of emergency logistics capability for public health emergencies: perspective of COVID-19. Int J Log Res Appl. (2022) 25:1509–22. doi: 10.1080/13675567.2021.1914566

[27] Ou ZW Wang HY Jiang DL Lu BL Gan W Liang J. Emergency logistics. J *Chongqing Univ (Nat Sci Ed)*. (2004) 3:164–7.

[28] Kołodziejczyk P Szołtysek J. Epistemology of social logistics. Organ Rev. (2009) 4:21–4.

[29] Xi MH. Emergency logistics management. Beijing: Emergency Management Press (2021) 2–3.

[30] Bao-de LI Jing LU Jing LI. Evaluation of emergency guarantee capability of key nodes in maritime transport based on improved copula function. J Transp Syst Eng Inf Technol. (2020) 20:20. Available at: http://www.tseit.org.cn/EN/Y2020/V20/I2/20

[31] Glick J. Enhancing logistics efficiency through computer and network technologies: a pathway to industry informatization. Acad J Sci Technol. (2024) 10:142–4. doi: 10.54097/e05gkp44

[32] Mangrulkar HS Samuel P Kumar P. Importance of supply chain & logistics post pandemic. EPRA Int J Econ Bus Manage Stud. (2022) 9:10–4.

[33] Malhotra G Mishra S. Effect of economic growth on the logistics sector in India. Theor Econ Lett. (2019) 9:210–22. doi: 10.4236/tel.2019.91016

[34] Hou R. Analysis of fixed asset investment benefits and their lag effects. Quant Econ Tech Econ Res. (2002) 3:13–6.

[35] Wang TY. Research on the lagged impact of fixed asset investment on economic growth in my country. Econ Issues. (2004) 12:50–2. doi: 10.16011/j.cnki.jjwt.2004.12.017

[36] Liang ZJ. An empirical test on the evolution of regional logistics development pattern. Stat Decis. (2017) 21:92–5. doi: 10.13546/j.cnki.tjyjc.2017.21.022

[37] Laboure M Taugourdeau E. Does government expenditure matter for economic growth? Global Pol. (2018) 9:203–15. doi: 10.1111/1758-5899.12540

[38] Ma SZ Guo JW. How does institutional innovation affect my country's cross-border e-commerce exports? ——empirical evidence from the establishment of comprehensive pilot zones. Manage World. (2022) 8:83–102. doi: 10.19744/j.cnki.11-1235/f.2022.0112

[39] Liang L Chen M Lu D. Revisiting the relationship between urbanization and economic development in China since the reform and opening-up. Chin Geogr Sci. (2022) 32:1–15. doi: 10.1007/s11769-022-1255-7

[40] Zhang K Liu XZ Shi JF. Trade openness, government size and economic growth. Macroecon Qual Res. (2018) 1:55–72. doi: 10.13948/j.cnki.hgzlyj.2018.3.005

[41] Hanif S Mu D Baig S Alam KM. A correlative analysis of modern logistics industry to developing economy using the VAR model: a case of Pakistan. J Adv Transp. (2020) 2020:1–10. doi: 10.1155/2020/8861914

[42] Fernald J Li H. Is slow still the new normal for GDP growth? FRBSF Econ Lett. (2019) 17:1–5.

[43] Harrison A Skipworth H van Hoek RI Aitken J. Logistics management and strategy. London: Pearson UK (2019) 168–9.

[44] Park S Park H. Analysis of regional specialization and value-added contribution of local logistics industry. J Korea Port Econ Assoc. (2020) 36:87–107. doi: 10.38121/kpea.2020.06.36.2.87

[45] He L. Several issues facing the promotion of high-quality development of logistics industry. China Circ Econ. (2018) 10:3–7.

[46] Chen H Wei JC Wei XF. Driving factors and sources of power for the development of China’s logistics industry—from the perspective of labor input. Bus Econ Manage. (2015) 11:13–26. doi: 10.14134/j.cnki.cn33-1336/f.2015.11.002

[47] Chen H Zhang Y. Research on the path of sustainable development of China’s logistics industry driven by capital factors. Sustain For. (2023) 15:297. doi: 10.3390/su15010297

